# Engineered iron oxide nanoplatforms: reprogramming immunosuppressive niches for precision cancer theranostics

**DOI:** 10.1186/s12943-025-02443-2

**Published:** 2025-09-01

**Authors:** Chao Yang, Shenglong Li, Liming Wang

**Affiliations:** 1 Trauma Center and Department of Burns, The First Hospital of China Medical University, Shenyang, China; 2 Second Ward of Bone and Soft Tissue Tumor Surgery, Cancer Hospital of Dalian University of Technology, Cancer Hospital of China Medical University, Liaoning Cancer Hospital & Institute, No. 44 Xiaoheyan Road, Dadong District, Shenyang, 110042 Liaoning China; 3 Department of Thoracic Surgery, The First Hospital of China Medical University, No. 155 Nanjingbei Street, Heping District, Shenyang, 110001, Liaoning China

**Keywords:** Iron oxide nanoparticles (IONPs), Immunotherapy, Tumor microenvironment reprogramming, Multimodal theranostics, Biohybrid nanoplatforms

## Abstract

Iron oxide nanoparticles (IONPs) have transitioned from conventional magnetic resonance imaging (MRI) contrast agents into structurally programmable combined imaging/treatment tools, leveraging their superparamagnetism, catalytic activity, and surface engineering versatility to achieve spatiotemporal control over drug delivery and immune modulation. Advances in nanofabrication now yield size-optimized aggregates with enhanced tumor accumulation through the enhanced permeability and retention (EPR) effect, while clinically approved formulations like ferumoxytol demonstrate intrinsic immunomodulatory functionality, positioning IONPs as pivotal tools for precision oncology. Conversely, cancer immunotherapy remains limited by the immunosuppressive tumor microenvironment (TME), where cellular suppression via M2-polarized macrophages and regulatory T cells (Tregs) synergizes with physical exclusion from dense extracellular matrices and metabolic sabotage through lactate-driven acidosis. These barriers establish “immune-cold” phenotypes characterized by deficient CD8⁺ T-cell infiltration and tertiary lymphoid structure formation, driving checkpoint inhibitor resistance with sub-30% response rates in solid tumors. To overcome these constraints, IONPs orchestrate multimodal immunotherapeutic strategies: they reprogram suppressive niches by polarizing macrophages toward M1 phenotypes, activate STING pathways, and induce immunogenic ferroptosis; enable precision delivery via magnetic lymph node targeting and cancer cell membrane-mediated homologous tumor homing; and facilitate real-time theranostics through MRI/magnetic particle imaging (MPI)-monitored immune cell trafficking. Preclinical validation confirms synergistic efficacy, with combinatorial regimens achieving over 50% complete tumor regression by converting immunologically cold microenvironments into inflamed states. This review systematically explores cutting-edge IONP-based innovations—spanning immune cell engineering, biohybrid systems, and energy-amplified therapies—that bridge localized tumor eradication with systemic antitumor immunity, while critically evaluating translational barriers for clinical implementation.

**Graphical abstract:**

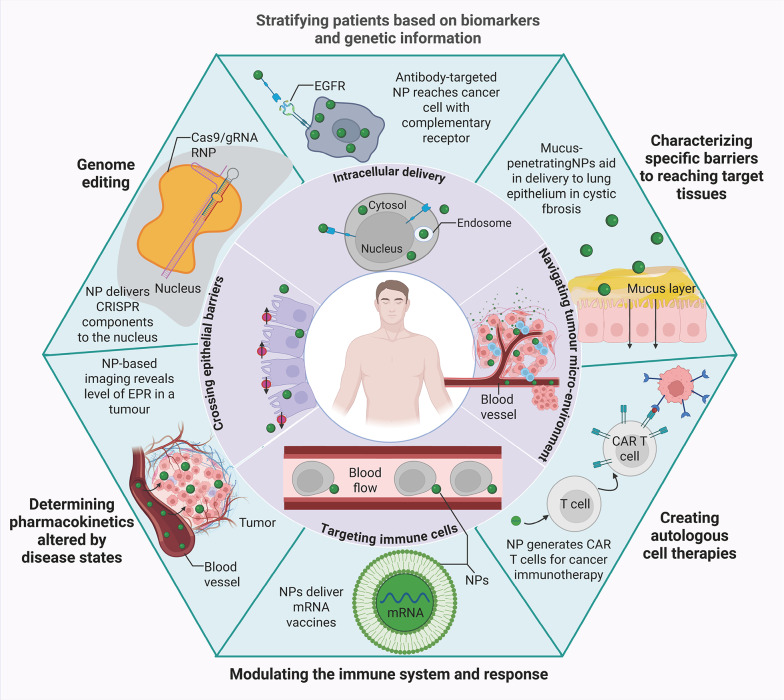

## Introduction

Iron oxide nanoparticles (IONPs) are a promising tool in oncological nanomedicine, characterized by their intrinsic superparamagnetism, biocompatibility, and ability to undergo flexible surface modifications [1–4]. Originally developed as magnetic resonance imaging (MRI) contrast agents—exemplified by clinically established formulations like ferumoxytol—IONPs have evolved into multifunctional combined imaging/treatment tools designed to precisely control where and when treatments act [3–7]. Their unique capabilities include magnetic navigation for enhanced tumor-targeted delivery, stimulus-triggered drug release activated by tumor microenvironment (TME)-related cues, and catalytic generation of cytotoxic species that simultaneously induce immunogenic cell death (ICD) [8–12]. New designs now boost clinical potential significantly: optimized tiny structures improve body distribution patterns while maintaining renal clearance capacity; surface modifications via biomimetic coatings significantly prolong systemic circulation; and enhanced magnetic responsiveness enables efficient energy conversion under clinically relevant conditions [3, 13–18]. Platelet membrane coatings further demonstrate active metastasis suppression through P-selectin-mediated circulating tumor cell capture [19]. Unlike conventional biomimetic coatings, recent IONP designs integrate dual-pathway metastasis interception—simultaneously capturing CTCs and clearing immunosuppressive exosomes—surpassing the single-mechanism targeting of earlier NanoGhosts platforms [20]. These developments collectively address historical challenges in tumor penetration and retention efficiency. Clinically, IONP-based systems demonstrate integrated diagnostic-therapeutic functionality. Ferumoxytol not only corrects iron deficiency but also modulates macrophage polarization in ongoing oncology trials, while blood-brain barrier-penetrating designs show marked efficacy in aggressive glioblastoma models [7, 21–25]. SPION-labeled cellular therapies further exemplify this convergence, enabling non-invasive monitoring of immune cell trafficking dynamics [26–29]. As precision oncology evolves, IONPs have emerged as indispensable biological tools that reconcile nanoscale engineering with therapeutic complexity, delivering superior targeting precision while mitigating systemic toxicity across preclinical and clinical applications [30–33].

The TME plays a critical role in shaping the immune response within solid tumors, orchestrating a complex network of immunosuppressive barriers [34–38]. This network is composed of biological, physical, and metabolic factors that work synergistically to suppress immune function [39–41]. Cellular suppression is predominantly mediated by M2-polarized tumor-associated macrophages (TAMs), myeloid-derived suppressor cells (MDSCs), and regulatory T cells (Tregs) [42–48]. These cells secrete immunosuppressive factors such as interleukin-10 (IL-10), transforming growth factor-β (TGF-β), and adenosine, which inhibit the activity of cytotoxic T lymphocytes and prevent dendritic cell maturation [49–54]. Additionally, the upregulation of immune checkpoint molecules such as PD-L1, CTLA-4, and TIM-3 exacerbates T-cell exhaustion, further contributing to immune evasion [35, 36, 55–57]. The TME is also characterized by several physical barriers, including a dense extracellular matrix comprised of components like collagen and hyaluronic acid [34, 58, 59]. These structural elements create interstitial pressures exceeding 40 mmHg, which impede the effective penetration of therapeutic agents and hinder immune cell infiltration [60, 61]. Moreover, abnormal tumor vasculature disrupts perfusion and promotes hypoxia, exacerbating the challenges faced by immune cells and therapeutic agents alike [62–64]. Metabolically, hypoxia-driven acidosis and lactate accumulation suppress the cytotoxic activity of natural killer (NK) and T cells, while nutrient competition further starves effector cells [65–68]. Together, these factors create a poorly immunogenic tumor state, characterized by low CD8^+^ T-cell infiltration and a lack of tertiary lymphoid structures, leading to response rates below 30% to checkpoint inhibitors in solid tumors [69–71].

IONPs offer a transformative solution to overcoming these barriers by integrating diagnostic, therapeutic, and immune-altering abilities. Their intrinsic ability to reprogram TAMs toward antitumor phenotypes, induce ICD, and deliver immune checkpoint inhibitors directly addresses the immunosuppressive mechanisms within the TME [27, 72, 73]. Furthermore, real-time monitoring via MRI and magnetic particle imaging enables the personalization of therapeutic strategies, allowing for dynamic adjustments based on treatment response [4, 74–76]. This review highlights cutting-edge IONP-based strategies-ranging from engineered immune cells and biomimetic systems to energy-triggered immunomodulation—that bridge localized tumor targeting with the activation of systemic antitumor immunity.

### Advancements in ionps for overcoming biological barriers in cancer immunotherapy and precision medicine

#### Overcoming biological barriers: the role of nanoparticles in precision medicine

Nanoparticles, particularly IONPs, are increasingly recognized for their potential to revolutionize cancer treatment. Their unique properties enable them to overcome numerous biological barriers that traditionally hinder the efficacy of conventional therapies, especially in oncology [77–79]. These barriers are multifaceted, encompassing challenges related to cellular uptake, immune system evasion, and precise tissue-specific delivery [80–82]. Conventional drugs often fail to penetrate cell membranes effectively due to their size and electrostatic repulsion, limiting their ability to target tumors with the required specificity [83, 84]. IONPs, on the other hand, can be designed to overcome these delivery challenges more accurately, enhancing the drug delivery process and improving the therapeutic index [12, 85, 86].

One of the key obstacles in cancer therapy is achieving efficient cellular uptake of therapeutic agents. Traditional drugs face challenges in crossing the cell membrane, due to size limitations and electrostatic repulsion [87, 88]. However, nanoparticles, particularly those modified with surface coatings, can overcome these hurdles. For example, IONPs can be engineered with specific surface modifications that improve cellular binding and facilitate internalization [89–91]. This can occur either passively, through the enhanced permeability and retention (EPR) effect, or actively, using targeting ligands that specifically bind to cell surface receptors [92, 93]. The ability of IONPs to selectively target tumor cells significantly reduces off-target effects, thereby improving the therapeutic outcome and minimizing toxicity to healthy tissues [94–96]. This enhanced targeting capability is particularly crucial in cancer therapy, where maximizing treatment efficacy while reducing side effects is paramount.

In addition to overcoming cellular uptake challenges, nanoparticles like IONPs also address the issue of immune system evasion. The immune system constantly monitors the body for foreign invaders, including therapeutic agents, which it neutralizes through processes such as phagocytosis [97–99]. This poses a significant challenge for the effective delivery of cancer therapies. To circumvent this issue, IONPs can be engineered to evade immune detection. Surface modifications, such as PEGylation, provide a “stealth” layer that prolongs the circulation time of the nanoparticles, reducing their clearance by the immune system [100–102]. This is particularly important for therapeutic nanoparticles that need to remain in circulation long enough to reach their target tissues. This extended circulation time is crucial for immunotherapies, which often require prolonged interaction with the immune system to achieve optimal therapeutic effects.

**Fig. 1:**
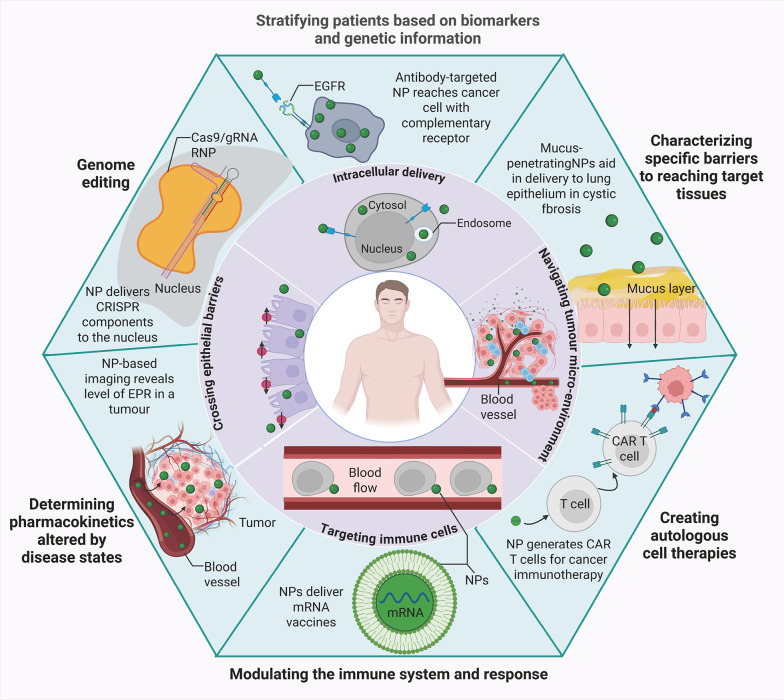
This schematic illustrates a dual-ring framework summarizing nanomedicine advancements. The inner ring denotes key biological barriers addressable by engineered nanoparticles—including cellular internalization hurdles, immune clearance evasion, and tissue-specific targeting—while the outer ring highlights precision medicine applications enhanced by these strategies. Nanoparticle platforms demonstrate capabilities to overcome these physiological constraints, thereby optimizing therapeutic delivery. Representative applications encompass chimeric antigen receptor (CAR) immunotherapies, epidermal growth factor receptor (EGFR)-targeted interventions, exploitation of the enhanced permeability and retention (EPR) effect, and CRISPR-based delivery of guide RNA (gRNA)/ribonucleoprotein (RNP) complexes. Collectively, these innovations expedite clinical translation of precision therapeutics

Moreover, IONPs can significantly improve targeted tissue delivery, a cornerstone of precision medicine [103–105]. One of the most important advantages of nanoparticles is their ability to accumulate preferentially in tumor tissues. This is due to the leaky vasculature typical of tumors, a phenomenon referred to as the EPR effect [106, 107]. However, while the EPR effect facilitates some degree of tumor targeting, it may not be sufficient for precise and effective treatment. Therefore, IONPs can be further engineered with specific targeting ligands that recognize overexpressed tumor markers, such as epidermal growth factor receptors (EGFR) or other cancer-specific antigens [108–110]. By ensuring that the nanoparticles selectively accumulate within the TME, therapeutic agents can be delivered directly to the site of disease, minimizing systemic exposure and reducing side effects. Overall, Fig. 1 illustrates a dual-ring framework summarizing nanomedicine advancements.

### Applications of nanoparticles in precision medicine

The integration of nanoparticles into precision medicine holds tremendous promise for improving cancer treatment outcomes. IONPs, in particular, offer unique capabilities that can dramatically enhance the efficacy of various cancer therapies. A prominent application of IONPs is in chimeric antigen receptor (CAR) therapies [111, 112]. CAR-T cell therapy has demonstrated remarkable success in treating hematological cancers by genetically modifying a patient’s T cells to express receptors that target tumor antigens. However, the delivery and activation of CAR-T cells within the body remains a significant challenge. IONPs can be used to deliver genetic material—such as mRNA or DNA encoding for CAR constructs—directly to T cells, ensuring efficient modification and improved anti-tumor activity [113]. Moreover, IONPs can be used to track the distribution of CAR-T cells and monitor their infiltration into tumors using MRI [114]. This provides valuable real-time information on treatment progress and enables dynamic adjustments to therapy, enhancing the overall effectiveness of CAR-T cell therapy.

Another significant application is the use of IONPs for targeted drug delivery, particularly to cancer cells overexpressing tumor-specific receptors. One of the most extensively studied targets is the EGFR, which is overexpressed in a variety of cancers, including non-small cell lung cancer (NSCLC) and glioblastomas [108, 115, 116]. By utilizing IONPs to deliver chemotherapeutic agents, immunomodulators, or RNA-based therapeutics—such as guide RNA (gRNA) and ribonucleoprotein (RNP) complexes—specifically to these tumors, it is possible to achieve higher local concentrations of the therapeutic agents [117–119]. This targeted delivery ensures that the treatment is highly effective, while minimizing systemic side effects that are often seen with conventional chemotherapy. Additionally, the application of external magnetic fields can further enhance the targeting of IONPs to the tumor site, ensuring greater precision in drug delivery and reducing the likelihood of off-target effects.

### Comparative analysis of ionps and alternative nanoplatforms

IONPs exhibit distinctive advantages for cancer immunotherapy that merit rigorous assessment relative to alternative nanotechnologies. IONPs demonstrate superior magnetic responsiveness enabling precise tumor targeting capabilities not achievable with non-magnetic platforms [10]. Their intrinsic catalytic activity facilitates reactive oxygen species (ROS)-dependent therapeutic mechanisms, including ferroptosis induction and chemodynamic modulation, outperforming gold nanoparticles in these fundamental processes [120].

In contrast, gold nanoparticles possess enhanced photothermal conversion properties that render them particularly suitable for photoimmunotherapy applications [121, 122]. Polymeric nanoparticles offer greater payload versatility and more predictable biodegradation kinetics, advantageous for sustained immunomodulator delivery [123, 124]. Lipid-based systems maintain dominance in nucleic acid delivery applications despite limited tumor-targeting specificity [125, 126].

Clinical translation landscapes reveal significant differentiation among platforms. IONPs lead in regulatory approvals for human use, though applications beyond iron supplementation remain investigational [4]. Gold nanoparticles show promise in localized therapies but exhibit delayed progress in systemic immunotherapeutic applications [127]. Polymeric and lipid platforms demonstrate established clinical utility for conventional drug delivery yet face challenges in achieving multifunctional immunotherapeutic integration comparable to IONPs [128, 129].

Therapeutic limitations further distinguish these technologies. IONPs present potential oxidative safety concerns requiring careful dosing optimization [75, 130]. Gold nanoparticles suffer from penetration depth restrictions in deep-seated malignancies [131]. Polymeric carriers may trigger batch-dependent immune reactions, while lipid systems demonstrate suboptimal extrahepatic biodistribution [132]. This technological ecosystem positions IONPs advantageously for magnetically guided immunotherapeutic strategies, with alternative platforms serving complementary roles in precision oncology [133].

## The potential roles of ionps in cancer immunotherapy

The potential of IONPs to address these critical challenges in cancer immunotherapy and precision medicine is vast. However, successful clinical translation of these technologies requires overcoming several key hurdles. One of the primary challenges is the scalability and reproducibility of nanoparticle production, which is essential for clinical use. Advances in nanomaterial synthesis, quality control, and manufacturing processes will be crucial to meet the stringent requirements for human use. Additionally, the long-term safety of nanoparticles must be rigorously evaluated, including potential toxicity, immune reactions, and biodistribution.

### IONPs: intrinsic activators of antitumor immunity

IONPs naturally stimulate immune responses, positioning them as promising agents for cancer immunotherapy. Intrinsic immune-altering abilities of IONPs were showed in Table 1. A notable example is ferumoxytol, a clinically approved formulation, which has demonstrated therapeutic efficacy in treating early-stage breast cancer and lung metastases. Ferumoxytol exerts its effect by polarizing TAMs toward a pro-inflammatory M1 phenotype. Systematic TAM reprogramming approaches follow in Sect. "Reprogramming TAMs via iron oxide nanoplatforms". In vitro studies have shown that ferumoxytol enhances macrophage production of reactive oxygen species (ROS) and augments cancer cell cytotoxicity. Furthermore, in vivo experiments have reported significant suppression of subcutaneous adenocarcinoma growth in murine models, with effects observed at both high and low concentrations, suggesting a dose-independent response [134]. Importantly, this tumor-suppressive effect appears to be independent of nanoparticle coating, as similar outcomes were observed with dextran-coated ferumoxtran-10 [134].

**Fig. 2:**
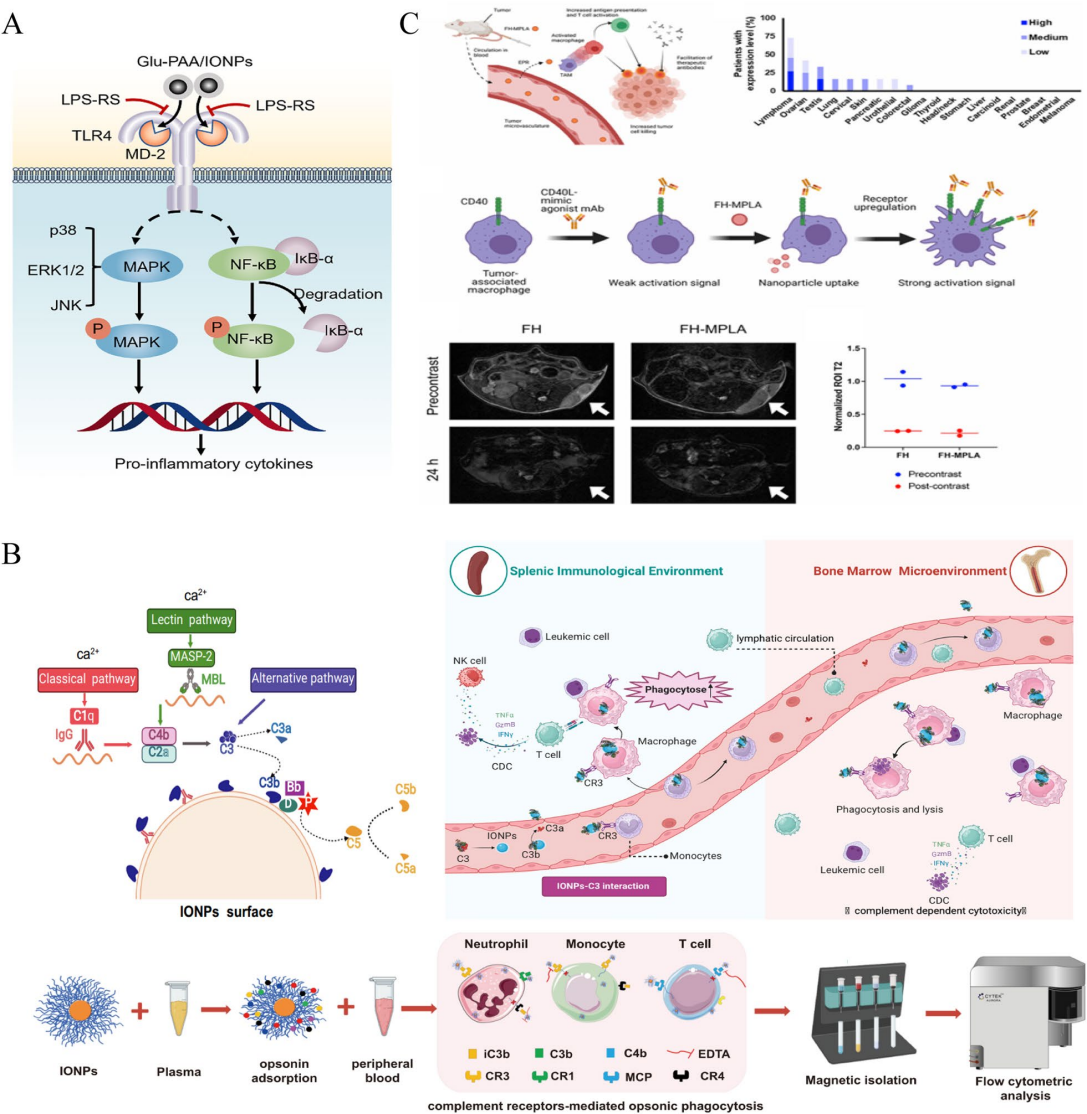
Iron oxide nanoparticles: intrinsic activators of antitumor immunity. **A** Schematic illustration: LPS-RS prevents the interaction of Glu-PAA/IONP with TLR4-MD2 and the activation of downstream signaling.Reproduced with permission [136]. Copyright from Elsevier Ltd, 2024. **B** Nanoparticles dynamically interact with immune cells via complement corona to exert antileukaemia efficacy.Reproduced with permission [2]. Copyright from Springer Nature, 2024. The interactions occurring at the IONP-complement interface are crucial in shaping the immunological outcomes. Iron oxide nanoparticles (IONPs) can opsonize with complement component C3b, subsequently engaging the C3 receptor (CR3) on circulating monocytes. These activated monocytes then migrate to immune organs and differentiate into resident macrophages. Concurrently, the IONP-C3b complex enhances the phagocytic activity of these resident macrophages against leukemia cells. Moreover, macrophages are capable of presenting tumor antigens to T cells, thereby activating adaptive anti-leukemia immunity. **C** Therapeutic scheme and imaging of nanoparticle deposition in tumors.Reproduced with permission [29]. Copyright from American Chemical Society, 2023. General framework for FH-MPLA immunotherapy in solid tumors. CD40 expression analyzed by IHC on cancer samples from human patients, archived in the Human Protein Atlas bioinformatics repository. CD40 expression is predominantly observed in lymphomas; most solid tumors exhibit negligible expression. Potential mechanism of FH-MPLA in augmenting α-CD40 therapy: FH-MPLA enhances CD40 expression on antigen-presenting cells, thereby amplifying activation by agonistic α-CD40 monoclonal antibodies. Representative T2-weighted MRI contrast images of mice pre- and post-injection with either unloaded FH or FH-MPLA nanoparticles. Tumor ROI indicated by white arrows. Quantification of T2 signal intensity within the tumor ROI, normalized to CSF signal intensity in pre- and post-contrast scans.

Beyond its clinical formulations, IONPs—especially ultrasmall Fe_3_O_4_ nanoparticles—act as potent immune enhancers when conjugated with model antigens like ovalbumin (OVA). The Fe_3_O_4_-OVA nanocomposite has been shown to stimulate dendritic cell maturation, promote T-cell activation, and engage macrophages. This composite effectively inhibits both subcutaneous and metastatic B16-OVA tumor growth in therapeutic and prophylactic settings [135]. The immunomodulatory properties of IONPs are further amplified through surface engineering. For instance, glucosylation of IONPs transforms them into powerful agonists of the TLR4-MD2 complex, surpassing lipopolysaccharide (LPS) in their ability to activate the NF-κB, MAPK, and STAT1 signaling pathways, which in turn drive M1 polarization and enhance antitumor immunity [136] (Fig. 2A).

**Table 1:** Intrinsic immune-altering abilities of ionps

Nanoparticle type	Cancer model	Immune mechanism	Key findings	Therapeutic outcomes	References
Ferumoxytol	Early-stage breast cancer, lung metastasis	Enhances macrophage ROS production	Coating-independent tumor growth suppression	Inhibits subcutaneous adenocarcinoma growth; potential “off-label” use to prevent metastasis	[134]
		Induces M1 macrophage polarization	Equivalent efficacy at high/low concentrations		
Fe₃O₄ NPs	B16-OVA melanoma (subcutaneous/metastatic)	Acts as “nanoimmunoenhancer”	First evidence of Fe₃O₄ immunostimulatory function beyond imaging/drug delivery	Prevents tumor formation; suppresses growth of established tumors	[135]
		Stimulates DC maturation, T-cell activation, macrophage activation			
FH-MPLA	Immunotherapy-resistant melanoma	Activates antitumor macrophage phenotype	No chemical modification of nanoparticles/drugs required	Synergizes with α-CD40 mAb to induce tumor necrosis/regression	[29]
(Ferumoxytol + MPLA)		Reshapes tumor-immune microenvironment			
Glycosylated IONPs	Solid tumors	Direct interaction with TLR4-MD2 complex	Pro-inflammatory potency exceeds LPS in specific conditions	Triggers M1 macrophage polarization; enhances antitumor immunity	[136]
		Activates NF-κB/MAPK/STAT1 pathways			
IONP-COOH	AML	Complement-dependent phagocytosis of tumor cells	Surface chemistry dictates immune effectiveness	Indirect tumor cell killing; no direct cytotoxicity	[2]
(Carboxymethyl dextran-coated)		Activates innate/adaptive immunity	IONP-NH₂/IONP-OH ineffective due to distinct protein corona		

*MPLA* monophosphoryl lipid A,* DC* dendritic cells,* LPS* lipopolysaccharide,* AML* acute myeloid leukemia,* IONP-COOH* carboxymethyl dextran-coated iron oxide nanoparticles,* IONP-NH₂* amino-modified iron oxide nanoparticles,* IONP-OH* hydroxyl-modified iron oxide nanoparticles

The immune response induced by IONPs is heavily influenced by their surface chemistry. For example, carboxymethyl dextran-coated IONPs (IONP-COOH) have been shown to indirectly inhibit the progression of acute myeloid leukemia (AML) by complement-dependent enhancement of macrophage phagocytosis, which triggers tumor antigen presentation and initiates adaptive immune responses. In contrast, amino or hydroxyl-modified IONPs show limited efficacy, likely due to the divergent protein corona formation on their surfaces, which affects immune recognition [2] (Fig. 2B). This chemical specificity offers a strategic framework for designing nanoplatforms with optimized immunostimulatory capabilities.

In terms of clinical translation, ferumoxytol-loaded monophosphoryl lipid A (FH-MPLA) has been developed to reprogram macrophages toward an antitumor phenotype. When combined with α-CD40 monoclonal antibody therapy, FH-MPLA induces tumor necrosis and regression in melanoma models that are resistant to conventional immunotherapy. This combination strategy demonstrates dual targeting of both adaptive and innate immune systems without requiring chemical modifications, underscoring its translational potential for clinical application [29] (Fig. 2C).

### Multifunctional iron oxide platforms for coordinated Immunomodulation

Advanced IONP-based platforms have emerged as sophisticated vehicles for the co-delivery of therapeutic agents, enabling real-time imaging and targeted tumor accumulation. These systems leverage engineered nanostructures to overcome barriers in drug delivery while orchestrating multimodal antitumor responses. Clinically oriented designs often incorporate magnetic navigation to minimize off-target effects. Table 2 summarized the multifunctional iron oxide platforms for coordinated immunomodulation. For instance, IO@FuDex3 nanomedicine, which integrates T-cell activators with anti-PD-L1, has significantly extended median survival in preclinical models, while also reducing adverse events compared to soluble checkpoint inhibitors [137].

**Table 2:** Multifunctional iron oxide platforms for coordinated Immunomodulation

Nanoparticle system	Cancer model	Core components	Delivery strategy	Key mechanisms	Therapeutic outcomes	References
Zn-doped IONPs + TLR agonists	Aggressive melanoma	Zn-doped Fe₃O₄ MNPs	MRI-trackable vaccine delivery	Synergistic T-cell activation	Significant tumor suppression in aggressive melanoma	[141]
		PolyIC (TLR3 agonist)	Immune checkpoint blockade	Enhanced tumor-specific immunity		
		R837 (TLR7 agonist)				
		OVA antigen				
		Anti-PD-L1/PD-1				
IO@FuDex3	Solid tumors	SPIONs + fucoidan/aldehyde dextran	Magnetic navigation to tumor site	Tumor immune escape inhibition	Adverse events; median survival vs. soluble anti-PD-L1	[137]
		Anti-PD-L1	Minimized off-target effects	TIL reactivation		
		Anti-CD3/CD28 (T-cell activators)				
MIRDs	Broad applicability	Fe₃O₄ MPs + ICG (core)	Long circulation	PTT-triggered tumor ablation	Synergistic PTT/immunotherapy inhibits growth/metastasis/recurrence	[142]
		DPA-PEG + R837 (shell)	MRI-guided magnetic targeting	R837-enhanced immune response		
SPIO NP@M-P	Lung cancer	SPIO NPs wrapped in H460 membrane	Homologous tumor targeting	PD-L1 blockade	TPP1 half-life 60; suppresses tumor growth in vitro/vivo	[139]
		TPP1 (PD-L1 inhibitor)	MMP2-triggered release	T-cell reactivation		
		PLGLLG (MMP2 substrate)				
Fe₃O₄-ICG@IRM	Ovarian cancer	Fe₃O₄-ICG core	Homologous targeting	PTT-induced antigen release	Effective against primary/metastatic tumors	[140]
		Hybrid ID8/RBC membrane (IRM)	Prolonged circulation	Enhanced antitumor immunity		
Fe₃O₄ core NP	Triple-negative breast cancer	Fe₃O₄ core	Tumor-specific targeting	Direct tumor killing	Tumor growth/metastasis; survival in aggressive model	[191]
		Doxorubicin	DC-mediated immune activation	Innate/adaptive immune activation		
		Poly(I: C) (TLR3 agonist)				
Magnetic silica-PLGA hybrid	Broad applicability	pH-sensitive carrier	pH-responsive drug release	PD-L1 gene silencing	Tumor growth vs. monotherapy; tumor cell apoptosis	[143]
		Paclitaxel (PTX)	Enhanced cellular uptake	CD8⁺ T-cell-mediated cytotoxicity		
		PD-L1 siRNA				
GOx@FeNPs	Colorectal cancer	Fe₃O₄ core/PDA shell	Dual tumor targeting	GOx-enhanced ferroptosis	> 90% tumor suppression with anti-PD-L1 synergy	[12]
		GOx	NIR-triggered PTT	PTT-induced ICD DC/CTL activation		
		PEG/cRGD/AA (dual targeting)				

*MNPs* magnetic nanoparticles, *TIL* tumor-infiltrating lymphocytes, *PTT* photothermal therapy, *ICG* indocyanine green, *RBC* red blood cell, *cRGD* cyclic arginylglycylaspartic acid, *AA* ascorbic acid, *GOx* glucose oxidase, *ICD* immunogenic cell death, *DC* Dendritic cells, *CTL* Cytotoxic T lymphocytes

**Fig. 3:**
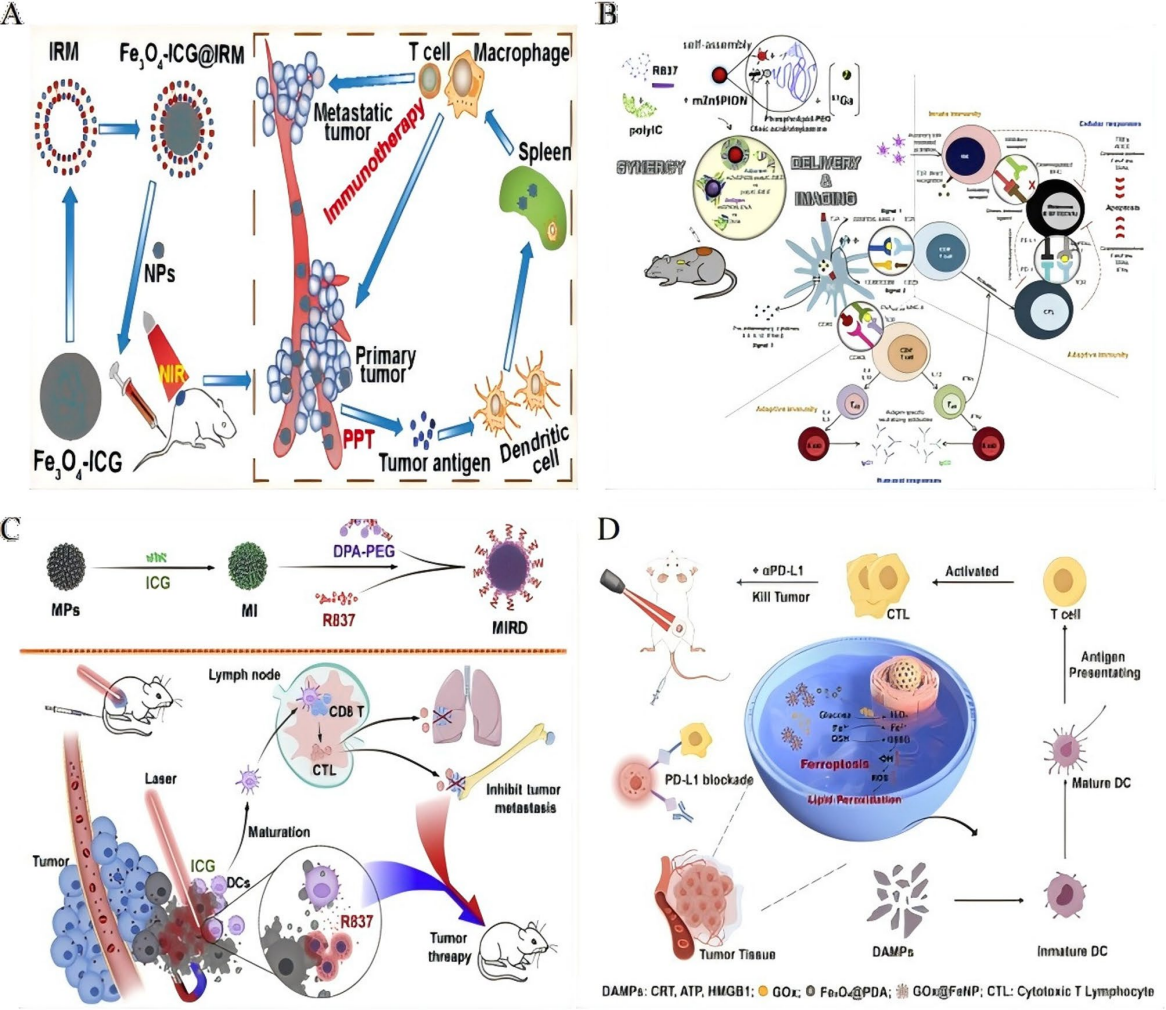
Multifunctional iron oxide platforms for coordinated immunomodulation. **A** The biomimetic Fe_3_O_4_-ICG@IRM nanoparticles showed synergistic photothermal-immunotherapy for ovarian cancer.Reproduced with permission [140]. Copyright from American Chemical Society, 2021. **B** Self-assembly achieved by simple physical mixture of the vaccine and nanovaccine components promotes effective drug synergy, delivery and imaging. Adjuvant and tumour antigen uptake by DCs at site of injection and/or via direct trafficking to LNs is able to activate potent and longlasting immunity against the B16-F10(OVA) melanoma cells, in particular when the expression of PD-L1 is downregulated.Reproduced with permission [141]. Copyright from Elsevier Ltd, 2018. **C** Once targeting to the tumor, the MIRDs with the near-infrared (NIR) irradiation caused tumor ablation and resulted in tumorassociated antigens releasing to induce the body's immunological response, which was markedly improved it to attack the tumors with the R837 releasing from the outer DPA-PEG.Reproduced with permission [142]. Copyright from Elsevier Ltd, 2020. **D** Schematic illustration of Gox@FeNPs-mediated PTT synergizing with ferroptosis by inducing ICD to improve colorectal cancer immunotherapy.Reproduced with permission [12]. Springer Nature, 2024.

Bioinspired targeting strategies further enhance tumor specificity. Current IONP-lipid hybrid systems achieve > 15% higher drug loading efficiency than mesoporous silica/carbon quantum dots (MSNP-CQD) while maintaining redox-responsive release, enabling real-time MRI-guided chemo-immunotherapy unattainable with static MSNP-CQD platforms [138]. For example, SPIO NP@M-P nanoparticles, cloaked in H460 lung cancer cell membranes and conjugated with a PD-L1-inhibitory peptide, prolonged the peptide half-life by 60-fold compared to free counterparts. This prolonged release was achieved through an MMP2-cleavable substrate, which effectively reactivated T cells and contributed to enhanced therapeutic efficacy [139]. Similarly, Fe₃O₄-ICG@IRM platforms coated with hybrid membranes derived from ID8 ovarian cancer cells and erythrocytes demonstrated prolonged circulation times and homologous targeting. These platforms enabled photothermally triggered antigen release, contributing to suppression of metastatic tumor growth [140] (Fig. 3A).

Beyond individual modifications, composite systems can synergize immunomodulation with physical energy-based therapies. For instance, zinc-doped IONPs conjugated with TLR agonists co-delivered PolyIC and R837, activating complementary innate immune pathways. When combined with PD-1/PD-L1 blockade, this platform elicited robust tumor-specific T-cell responses against aggressive melanoma [141] **(**Fig. 3B**)**. Another example, the MIRDs platform, co-loaded indocyanine green (ICG) and R837 into Fe₃O₄@DPA-PEG nanoparticles. Near-infrared (NIR) irradiation induced photothermal ablation and antigen release, while R837 enhanced antitumor immunity, leading to suppression of tumor recurrence [142] (Fig. 3C).

More complex composite systems, such as pH-responsive silica-PLGA hybrids, have been developed to co-encapsulate paclitaxel and PD-L1-targeting siRNA. These systems achieved sustained drug release and enhanced CD8⁺ T-cell-mediated tumor killing, improving overall therapeutic efficacy [143]. For metabolic modulation, GOx@FeNPs, which combine glucose oxidase (GOD)-enhanced ferroptosis with photothermal ICD and anti-PD-L1 blockade, have demonstrated impressive tumor suppression in colorectal cancer models. This outcome was achieved through dendritic cell maturation and cytotoxic T lymphocyte infiltration [12] (Fig. 3D).

### Engineering immune cells with iron oxide nanoplatforms

**Fig. 4:**
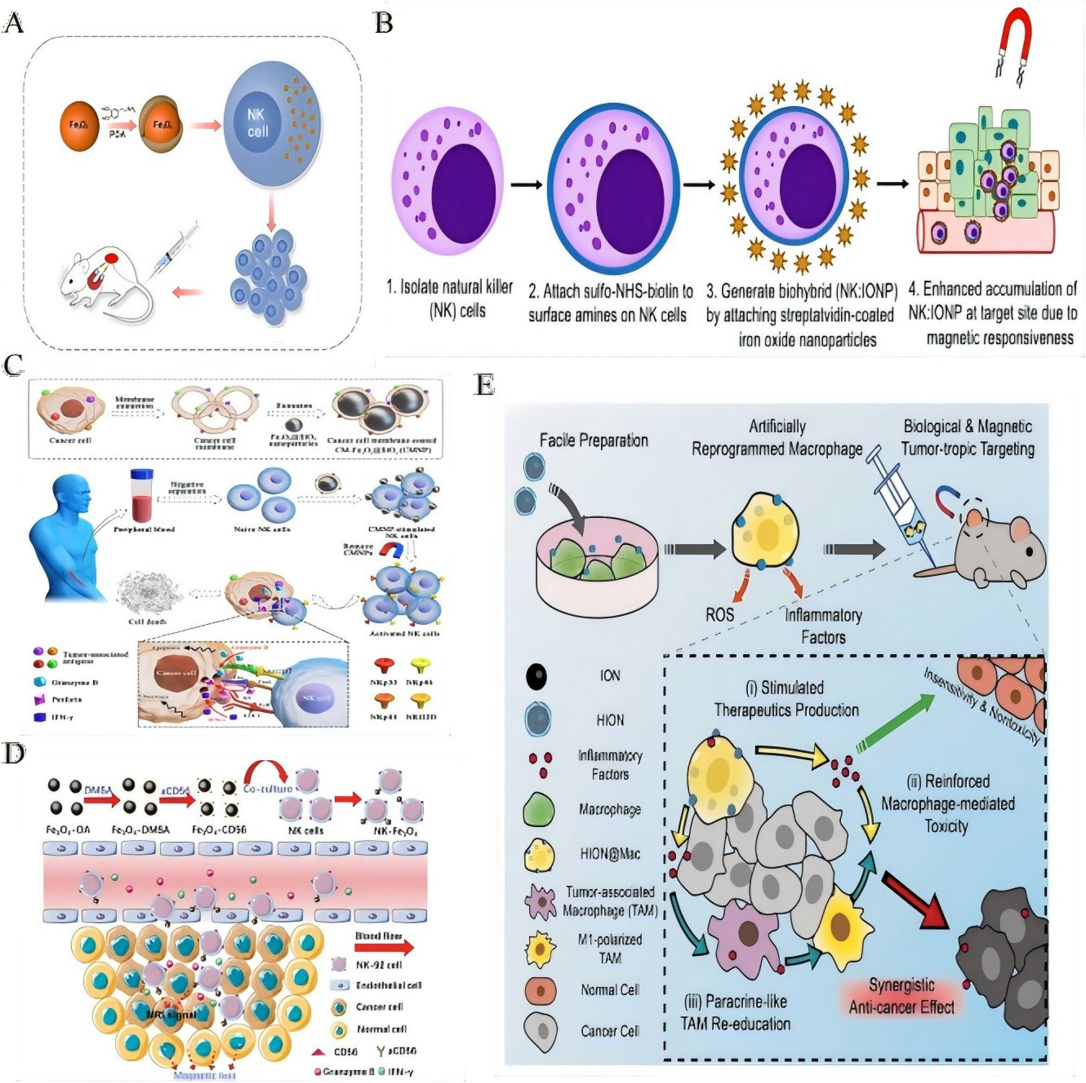
Engineering immune cells with iron oxide nanoplatforms. **A** Diagram illustrating the complete 3D molecular model used in the experiment.Reproduced with permission [144]. Copyright from Royal Society of Chemistry 2018. **B** Schematic illustration: designing magnetically responsive biohybrids composed of cord blood-derived natural killer cells and iron oxide nanoparticles.Reproduced with permission [145]. Copyright from American Chemical Society, 2019. **C** Schematic illustration: Cell membrane-encapsulated magnetic nanoparticles for enhancing natural killer cell-mediated cancer immunotherapy.Reproduced with permission [146]. Copyright from Elsevier Ltd, 2021. **D** A diagrammatic representation of the mechanism by which magnetically targeted NK-92 cells, in conjunction with iron oxide nanoparticles, suppress the proliferation of solid tumors. (DMSA: meso-2,3-dimercaptosuccinic acid and aCD56: anti-CD56 antibody).Reproduced with permission [147]. Copyright from Royal Society of Chemistry 2021. **E** The schematic representation illustrates that the engineered HION@Macs homing to tumors via active chemotaxis and magnetic navigation, secrete pro-inflammatory mediators (including TNF-α, NO, and ROS) to inhibit tumor growth, and reprogram in situ M2 macrophages into a pro-inflammatory M1 phenotype, facilitating a synergistic, cancer-specific therapeutic approach.Reproduced with permission [150]. Copyright from Wiley-VCH GmbH, 2019.

The strategic integration of IONPs with immune effector cells enables precise spatiotemporal control over tumor targeting and therapeutic efficacy (Table 3). NK cells, known for their intrinsic antitumor activity, are particularly promising candidates for such engineering approaches. Magnetic delivery systems utilizing Fe₃O₄@PDA nanoparticles have been shown to enhance the recruitment of NK cells to NSCLC tumors under the influence of an external magnetic field. This targeted approach significantly increases tumor cell apoptosis without impairing NK cell functionality [144] (Fig. 4A). In a similar vein, umbilical cord-derived NK cells conjugated with IONPs, referred to as NK: IONP hybrids, maintain their phenotype while demonstrating enhanced cytolytic activity in both 2D and 3D tumor models, showcasing the potential of magnetically guided precision therapy [145] (Fig. 4B).

**Table 3:** Engineering immune cells with iron oxide nanoplatforms

Immune cell type	Nanoparticle system	Engineering strategy	Functional modifications	Key mechanisms	Therapeutic outcomes	References
NK cells	Fe_3_O_4_@PDA NPs	Magnetic core-dopamine shell internalization	Enhanced tumor infiltration	Magnetic guidance under external field	Apoptosis of NSCLC cells; tumor growth	[144]
(Primary)			Unaltered biological function			
NK cells	IONP-conjugated	Surface conjugation via biocoupling	Preserved phenotype/function	Magnetic localization	Cytolytic activity in 2D/3D models	[145]
(Umbilical cord-derived)			Magnetically navigable			
Macrophages	HA-coated SPIONs	Internalization of HIONs	Tumor targeting	Reprogramming TAMs to M1 phenotype	Synergistic tumor suppression	[150]
(Patient-derived)	(HIONs)		ROS/cytokine production			
			Resistance to immunosuppressive TME			
T lymphocytes	SPION Citrate	Surface functionalization	Blood-compatible	Magnetic enrichment in “cold” tumors	T cell attraction by external magnetism	[148]
			Magnetic responsiveness			
NK cells	Fe_3_O_4_@SiO_2_ CMNPs	Cancer cell membrane coating	Activation receptor expression	Tumor antigen presentation	Enhanced antitumor capability of NK cells	[146]
			Cytotoxic factor secretion			
NK-92 cells	Anti-CD56-Fe_3_O_4_ conjugates	Antibody-mediated conjugation	Magnetically drivable migration	MRI-trackable targeting	Tumor suppression in solid tumors	[147]
Human T cells	SPION-loaded	Surface loading	Unaltered cell mechanics	Magnetic enrichment without functional impairment	Retained tumor-killing ability post-loading	[149]
(Incl. CAR-T)			Preserved proliferation/cytotoxicity			
T cells/CAR-T cells	Ultrasmall IONPs	Surface labeling	Unaffected viability/proliferation	MRI-based spatial distribution monitoring	Predictive of therapy response via tumor infiltration	[114]
(Human & murine)			Unimpaired effector function			
CAR-T cells	SPION-loaded	Surface loading	Cytokine release	Reduced systemic toxicity	Retained specific cytolytic activity; MRI-detectable	[111]
			Shift from pyroptosis to apoptosis			

*PDA* polydopamine,* NSCLC* non-small cell lung cancer,* HIONs* hyaluronic acid-coated superparamagnetic iron oxide nanoparticles,* TME* tumor microenvironment,* CMNPs* cancer cell membrane-coated nanoparticles,* CAR-T* chimeric antigen receptor T-cell,* SPION* superparamagnetic iron oxide nanoparticle

Bioinspired targeting strategies further enhance the therapeutic potential of these engineered cells. For instance, coating Fe₃O₄@SiO₂ nanoparticles with cancer cell membranes (CMNPs) enables the presentation of tumor-specific antigens to NK cells, thereby boosting the expression of activation receptors and enhancing cytotoxic factor secretion [146] (Fig. 4C). In the context of clinical translation, anti-CD56-conjugated Fe₃O₄ nanoparticles have been used to magnetically direct NK-92 cells to solid tumors. This approach not only facilitates targeted tumor suppression but also enables real-time MRI tracking, offering a non-invasive means of monitoring therapeutic outcomes [147] (Fig. 4D).

Similarly, T lymphocytes can benefit from magnetic functionalization, which enhances their targeting and efficacy. SPION-citrate-labeled T cells retain unimpaired viability and can be efficiently guided to immune-cold tumors via external magnetic fields, enhancing the effectiveness of checkpoint inhibitor therapy [148]. Notably, the loading of SPIONs on T cells preserves their proliferative capacity, tumor-killing ability, and functional integrity, which is crucial for the success of CAR-T cell therapies [149]. Ultrasmall IONP-labeled CAR-T cells retain their effector function while enabling MRI-based monitoring of tumor infiltration patterns, thus providing predictive insights into therapy response [114]. Interestingly, SPIONs have been shown to modulate CAR-T cell behavior by shifting the mode of tumor cell death from pyroptosis to apoptosis. This shift reduces the release of inflammatory cytokines and mitigates systemic toxicity, all while preserving cytolytic specificity [111].

Beyond effector cells, macrophages engineered with hyaluronic acid-coated SPIONs (HION@Macs) exhibit enhanced tumor-tropic properties, resistance to immunosuppression, and the ability to reprogram TAMs into an antitumor M1 phenotype. This reprogramming induces synergistic tumor suppression, further highlighting the potential of magnetically enhanced immune cells for comprehensive cancer therapy [150] (Fig. 4E).

### Physical energy-amplified immunotherapy via iron oxide nanoplatforms

**Fig. 5:**
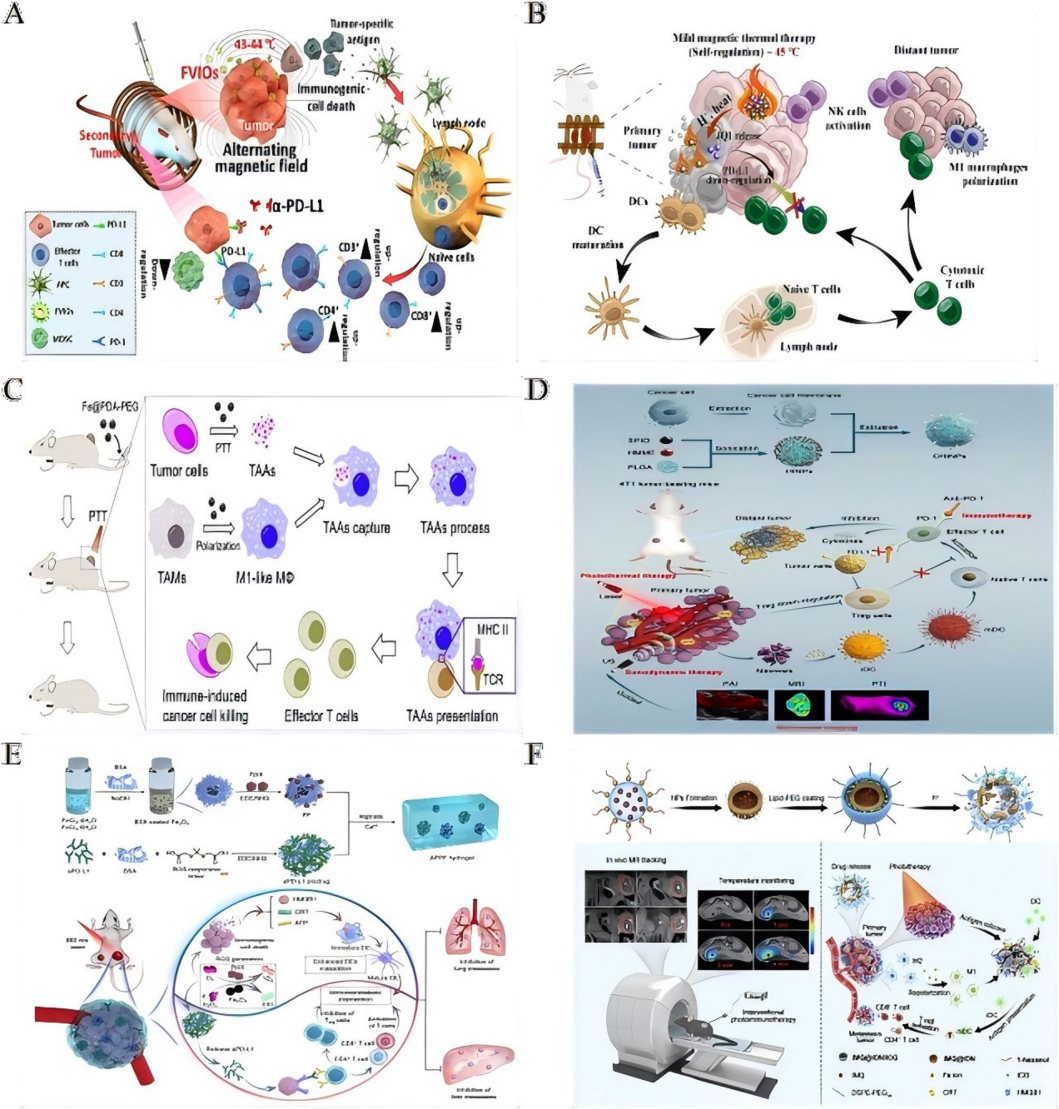
Physical energy-amplified immunotherapy via iron oxide nanoplatforms. **A** Ferrimagnetic vortex-domain iron oxide nanoring (FVIO)-mediated mild magnetic hyperthermia can activate the host immune systems and efficiently cooperate with PD-L1 blockade to inhibit the potential metastatic spreading as well as the growth of distant tumors.Reproduced with permission [151]. Copyright from American Chemical Society, 2019. **B** Schematic illustration of AMF triggered disassembly of JQ1/IONAs for self-regulated magnetic thermal therapy and controlled JQ1 release to improve immunological response.Reproduced with permission [152]. Copyright from Elsevier Ltd, 2023. **C** Fe@PDA-PEG induced M2-like TAMs to M1 polarization combined with PTT-induced TAAs release altering the functional orientation of the tumorpromoting microenvironment toward an antitumor mode with immune-induced cancer cell killing.Reproduced with permission [155]. Copyright from Elsevier Ltd, 2019. **D** PTT and SDT are synergistically augmented by a novel multimodal imaging nanoprobe integrated with cancer cell membrane-biomimetic nanoparticles (CHINPs) loaded with superparamagnetic iron oxide (SPIO) and hematoporphyrin monomethyl ether (HMME).Reproduced with permission [156]. Copyright from Springer Nature, 2022. CHINPs exhibit excellent homologous tumor targeting, and are sequentially triggered by ultrasound and near infrared (NIR) light under the guidance of magnetic resonance, photoacoustic and photothermal imaging, leading to complete in situ tumor eradication and systemic anti-tumor immune activation. Further combination of this approach with immune checkpoint blockade therapy is shown to suppress tumor metastasis. **E** Schematic illustration of the fabrication and applications in synergistic cancer immunotherapy of prodrug hydrogels. The fabrication illustration of prodrug hydrogels and the prodrug hydrogel-mediated synergistic PDT/CDT/immunotherapy to treat primary and distant tumors and prevent lung and liver metastasis.Reproduced with permission [158]. Copyright from Elsevier Ltd, 2022. **F** Schematic illustration of IMQ@IONs/ICG-mediated interventional photothermal immunotherapy for the treatment of metastatic pancreatic cancer. Schematic diagram of interventional photothermal-immunotherapy with imaging guidance and temperature monitoring by magnetic resonance (MR) technique and mechanism of anti-tumor immune response induced by IMQ@IONs/ICG based interventional photothermal-immunotherapy.Reproduced with permission [161]. Copyright from Elsevier Ltd, 2021.

The integration of physical energy modalities with immunotherapeutic agents, via iron oxide-based platforms, enables precise spatiotemporal control and enhances antitumor immune responses **(**Table 4**)**. Among these, magnetic hyperthermia has emerged as a potent immunogenic trigger. Ferromagnetic vortex-domain iron oxide nanorings (FVIOs) induce mild thermal stress, leading to the exposure of calreticulin (CRT) on tumor cells. This exposure initiates ICD, macrophage polarization, and CD8⁺ T-cell infiltration. When integrated with PD-L1 blockade (initially discussed in Sect. ''Multifunctional iron oxide platforms for coordinated immunomodulation''), FVIO-mediated hyperthermia significantly suppresses tumor recurrence and metastasis [151] (Fig. 5A). Similarly, temperature-responsive iron oxide nanoparticle assemblies (IONAs), loaded with the immunomodulator JQ1, deliver controlled heat and modulator release under alternating magnetic fields. This strategy effectively downregulates PD-L1 expression and eradicates primary tumors while preventing distal tumor growth [152] (Fig. 5B).

**Table 4:** Physical energy-amplified immunotherapy via iron oxide nanoplatforms

Nanoparticle system	Physical energy	Cancer model	Core components	Key mechanisms	Therapeutic outcomes	References
Ce6/Fe_**3**_**O**_**4**_**-L**	Ultrasound (SDT)	Solid tumors	Fe_3_O_4_ NPs (10 nm)	ROS-triggered Fe_3_O_4_ release	Eliminates SDT-treated & distant untreated tumors with anti-PD-L1	[157]
			Chlorin e6 (Ce6)	ETA capture/transport to LNs		
FVIOs	Mild magnetic hyperthermia	Metastatic tumors	FVIOs	CRT exposure “eat me” signal	CD8^+^ T-cell infiltration; recurrence/metastasis with PD-L1 blockade	[151]
				ICD & macrophage polarization		
MINPs	NIR-triggered PTT	Metastatic tumors	SPIO NPs	PA/MR imaging-guided ablation	Attacks residual/distant metastases	[162]
			CpG ODN	TAAs release systemic immunity		
Fe@PDA-PEG	NIR-triggered PTT	Colon/breast cancer	Polydopamine-coated iron-chelated NPs	M2→M1 TAM repolarization	Tumor volume; survival	[155]
				TAA presentation & T-cell infiltration		
TSIO	NIR-II-triggered PTT	Metastatic tumors	2D TiS_2_ nano-carriers	Magnetic targeting tumor accumulation	Recurrence/metastasis with PD-1 blockade	[153]
HCSVs	Circularly polarized MF	Solid tumors	IONCs	MF-triggered Fe^2+^ release Fenton reaction	CRT exposure antitumor immunity	[154]
			Ascorbic acid core			
Anti-HER2 SPIONs	Immunohyperthermia	HER2^+^ cancer	Antibody-conjugated SPIONs	Selective induction of apoptosis in HER2^+^ cells	Targeted elimination of antigen-expressing cells	[160]
CHINPs	PTT/SDT	Breast cancer	SPIO NPs	Sequential US/NIR activation complete tumor ablation	Systemic antitumor immunity; metastasis	[156]
			HMME			
			Cancer cell membrane			
GAPFBD	NIR-triggered PTT	Solid tumors	AuNCs/Fe_3_O_4_	pH-sensitive NO release + IDO inhibition	Long-term antitumor immunity	[218]
			GSNO/1-M-DT			
ROS-responsive hydrogel	PDT/CDT	Metastatic tumors	PpIX-Fe_3_O_4_	Enhanced ROS ICD + aPD-L1 activation	Primary tumor suppression & metastasis prevention	[158]
			ROS-responsive aPD-L1			
JQ1/IONAs	Mild magnetic hyperthermia	Metastatic tumors	Temp-responsive IONAs	Controlled JQ1 release + PD-L1 downregulation	Eradicates primary tumors; prevents recurrence	[152]
IMQ@IONs/ICG	MRI-guided PTT	Pancreatic cancer	IONs/ICG	Tumor penetration ICD	Complete primary tumor elimination; mesenteric metastasis	[161]
			Imiquimod			
IONP-C/O@LP	N/A (Vaccine)	Tumor models	IONPs conjugated to OVAp/CpG	Dual antigen/adjuvant delivery DC activation	Antigen-specific T-cell response; tumor suppression	[159]
			Lipid vesicles with P30 peptide			

*SDT* sonodynamic therapy, *CRT* calreticulin, *ODN* oligodeoxynucleotide, *HMME* Hematoporphyrin monomethyl ether, *IDO* indoleamine 2,3-dioxygenase, *aPD-L1* anti-PD-L1 antibody, *MF* magnetic field, *MINPs* magnetically responsive immunostimulatory nanoreagents, *PTT* photothermal therapy, *TAAs* tumor-associated antigens

Photothermal therapy (PTT) has shown robust synergy with immune checkpoint inhibition. Magnetically targeted TiS₂ nanocarriers, when subjected to NIR-II irradiation, generate tumor antigens that enhance the efficacy of PD-1 blockade, thus suppressing metastasis [153]. Additionally, hybrid vesicles (HCSVs) containing ascorbic acid and iron oxide nanocubes use circularly polarized magnetic fields to amplify Fenton reactions, generating ROS that promote ferroptosis-like cell death and CRT-mediated immunity [154]. Polydopamine-coated Fe@PDA-PEG nanoparticles combine PTT with macrophage polarization, enhancing M1 macrophage presentation of tumor antigens and T-cell infiltration, thereby extending survival in colon and breast cancer models [155] (Fig. 5C). Cancer cell membrane-camouflaged nanoplatforms (CHINPs), co-loaded with superparamagnetic iron oxide (SPIO) and sonosensitizers, enable sequential ultrasound-triggered sonodynamic therapy (SDT) and NIR-P-mediated PTT. This strategy achieves complete ablation of primary tumors and elicits systemic antitumor immunity [156] (Fig. 5D). Multimodal energy delivery systems significantly amplify immunostimulation through engineered responsiveness. For example, core-shell Ce6/Fe₃O₄-L nanoparticles, upon ultrasound exposure, release protein-capturing Fe₃O₄, which transports endogenous antigens to lymph nodes. This mechanism synergizes with anti-PD-L1 therapy to eliminate both treated and distant tumors [157] . ROS-responsive alginate hydrogels co-deliver protoporphyrin IX (PpIX)-modified Fe₃O₄ and anti-PD-L1 prodrugs. The resulting enhanced ROS generation induces ICD and activates checkpoint blockade, effectively suppressing both primary tumors and metastases [158] **(**Fig. 5E**)**. Additionally, the IONP-C/O@LP nanovaccine facilitates dual intracellular delivery of antigen and adjuvant to dendritic cells (DCs) via membrane fusion and endocytosis. By promoting ROS generation, IONPs enhance dendritic cell maturation, thereby eliciting potent T-cell responses [159].

Precision targeting of these platforms minimizes off-tumor toxicity. For instance, antibody-conjugated superparamagnetic iron oxide nanoparticles (SPIONs) targeting HER2 enable “immunohyperthermia” by selectively inducing apoptosis in antigen-expressing cancer cells in co-culture systems [160]. The tumor-microenvironment-sensitive IMQ@IONs/ICG platform improves drug penetration in pancreatic cancer, enabling MRI-guided interventional PTT. This strategy induces ICD, while imiquimod release triggers systemic immunity, leading to the eradication of primary tumors and reduction in metastases [161] (Fig. 5F). Lastly, magnetically responsive immunostimulatory nanoreagents (MINPs), co-loaded with SPIO and CpG oligodeoxynucleotides (ODNs), facilitate field-directed tumor accumulation for PTT. They release antigens that synergize with CpG to target residual and metastatic lesions [162].

### Ferroptosis and chemodynamic synergy in iron-enabled cancer immunotherapy

**Fig. 6:**
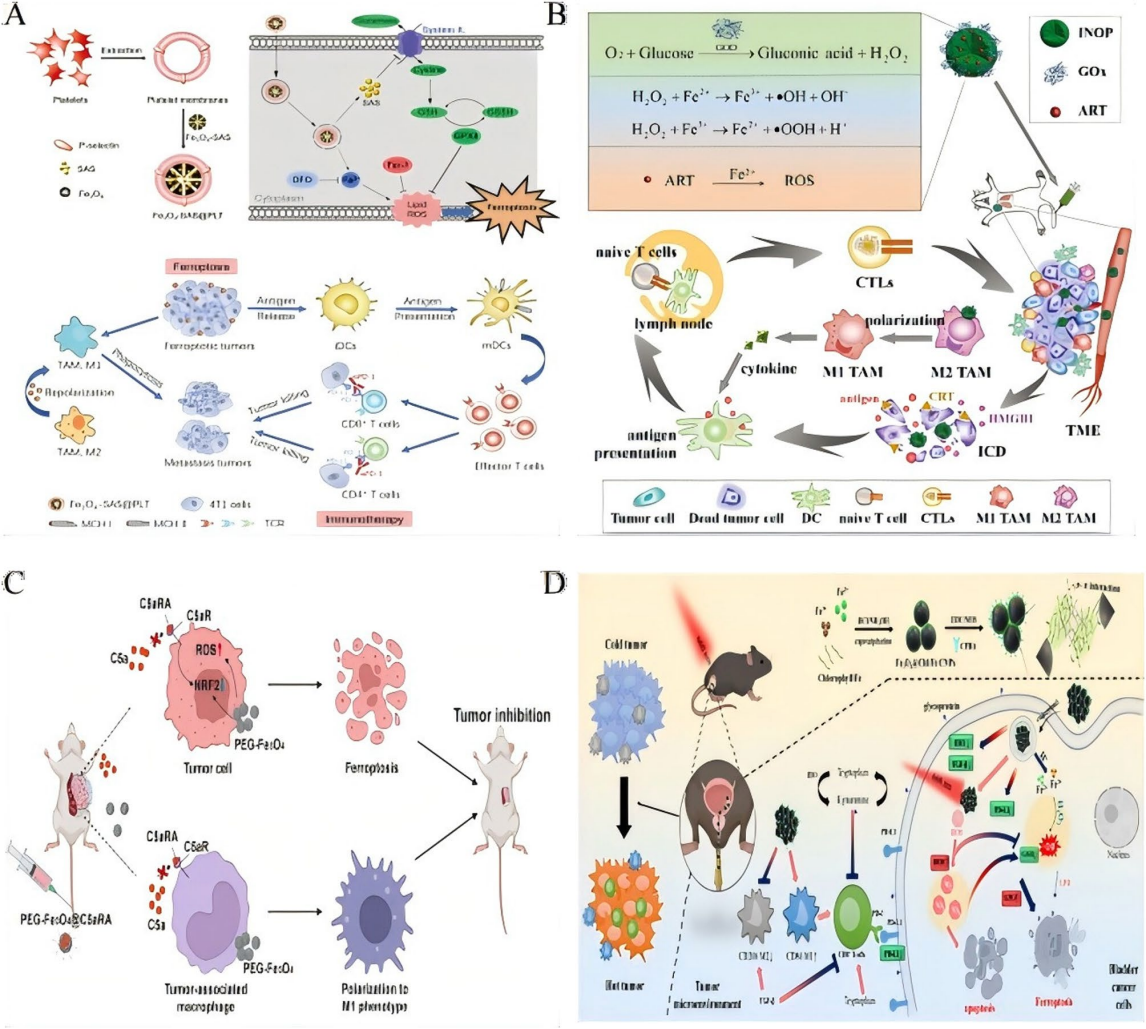
Ferroptosis and chemodynamic synergy in iron-enabled cancer immunotherapy. **A** Schematic illustration of platelet membrane-camouflaged magnetic nanoparticles for ferroptosis-enhanced cancer immunotherapy. Preparationof Fe_3_O_4_-SAS@PLT. Fe_3_O_4_-SAS@PLT-induced cell death by ferroptosis. Mechanisms of Fe_3_O_4_-SAS@PLT-mediated ferroptosis enhancing immune checkpoint blockade in metastasis tumors.Reproduced with permission [163]. Copyright from Wiley-VCH GmbH, 2020. **B** Schematic mechanism of IONP-GOD@ART for enhanced immunotherapy.Reproduced with permission [165]. Copyright from Wiley-VCH GmbH, 2021. **C** Schematic illustration of superior anti-tumor effect of PEG-Fe_3_O_4_@C5aRA through ferroptosis and macrophages polarization to M1 phenotype.Reproduced with permission [167]. Copyright from Frontiers Media SA, 2024. **D** Schematic illustration of the synthesis of Fe_3_O_4_@Chl/Fe-CPBA CNPs for targeted delivery into BC cells and demonstration of their antitumor effect and immunoregulatory effect within BC cells after the combination of chemodynamic and photodynamic therapy.Reproduced with permission [170]. Copyright from Springer Nature, 2022.

Following introduction of ferroptosis as an immunomodulatory mechanism (Sect. "Multifunctional iron oxide platforms for coordinated immunomodulation" applications), we now detail its biochemical foundations in reprogramming the tumor immune microenvironment (Table 5). Therapeutic exploitation of ferroptosis appears in Sect. "Biohybrid nanoplatforms: nature-inspired tumor immunotherapy" and "Discussion and prospects". Ferroptosis orchestrates T-cell infiltration through multistep mechanochemical signaling. *Mechanism I*: Ferroptotic cells release DAMPs (HMGB1, ATP) that activate dendritic cells via TLR4/P2 × 7 receptors, triggering CCR7-dependent migration to lymph nodes where they prime tumor-specific T cells. *Mechanism II*: Lipid peroxidation derivatives (e.g., 4-HNE, oxidized phospholipids) function as chemoattractants that: (i) upregulate endothelial VCAM-1/ICAM-1 to enable T-cell adhesion, (ii) generate CXCL9/CXCL10 gradients through STAT1 activation in stromal cells, and (iii) promote T-cell extravasation via CXCR3 binding. *Mechanism III*: Iron-catalyzed depletion of immunosuppressive Arg1^+^ MDSCs and M2-TAMs removes physical barriers and TGF-β/CD47 ‘don’t eat me’ signals, permitting T-cell penetration into tumor cores. These coordinated changes convert immune-excluded phenotypes into T-cell-inflamed microenvironments, potentiating checkpoint responses. Platelet-membrane-coated Fe₃O₄-SAS@PLT nanoparticles exploit immune evasion mechanisms to deliver sulfasalazine (SAS), thereby inducing ferroptosis that reprograms immunosuppressive M2 macrophages into antitumor M1 phenotypes. This reprogramming enhances the efficacy of PD-1 blockade in metastatic breast cancer by triggering tumor-specific immunity [163] (Fig. 6A). Similarly, chemically programmed iron oxide nanovaccines (IONVs) combine catalytic iron delivery with targeted antigen presentation, eradicating aggressive tumors through ferroptosis-driven danger signals and the repolarization of TAMs into M1 phenotypes [164].

Metabolic modulation within the TME significantly amplifies therapeutic efficacy. The IONP-GOD@ART cascade platform utilizes GOD to deplete glucose and generate hydrogen peroxide (H₂O₂), fueling Fenton reactions that produce hydroxyl radicals for chemodynamic therapy (CDT). Concurrently, artemisinin (ART)-derived ROS polarize M2 TAMs to M1 states, creating a “butterfly effect” that drives tumor regression and prevents metastasis [165] (Fig. 6B). In a similar approach, Lp-IO liposomes enhance intraliposomal Fenton reactions, promoting lipid peroxidation and ferroptosis while enabling pH/ROS-responsive release of doxorubicin, synergistically increasing cytotoxicity [120].

**Table 5:** Ferroptosis and chemodynamic synergy in iron-enabled cancer immunotherapy

Nanoparticle system	Cancer model	Key components	Mechanism of action	Combined therapy	Therapeutic outcomes	References
Fe_3_O_**4**_-SAS@PLT	Metastatic 4T1 breast cancer	Fe_3_O_4_	Platelet membrane enables immune evasion/tumor targeting	Anti-PD-1	Anti-PD-1 efficacy; triggers tumor-specific immunity	[163]
		SAS	SAS induces ferroptosis ICD			
		Platelet membrane	M2 M1 macrophage repolarization			
IONVs	Aggressive tumors	Chemically programmed iron oxide core	Catalytic iron delivery ferroptosis	Antibody-mediated TME modulation	Complete tumor eradication; durable antitumor immunity	[164]
			Targeted antigen delivery via reversible covalent bonds			
			Reprograms TAMs to antitumor state			
IONP-GOD@ART	Solid tumors	Mesoporous IONPs	GOD depletes glucose → H_2_O_2_ generation	N/A	“Butterfly effect”: tumor regression & metastasis prevention	[165]
		GOD	Fe^2+^/Fe^3+^ Fenton reaction OH			
		ART	ART-derived ROS → ICD			
			M2→M1 TAM polarization			
Fe_3_O_**4**_@Chl/Fe CNPs	Bladder cancer (BC)	Fe_3_O_4_ clusters	Intravesical CPBA-targeted delivery	PDT + CDT	Survival; remodels immunosuppressive TME	[170]
		Chlorin photosensitizer	PDT/CDT synergy ROS/ferroptosis			
Lp-IO	Broad applicability	PEGylated IONPs embedded in liposomes	Fenton reaction in lipid bilayer → lipid peroxidation	Doxorubicin chemotherapy	Synergistic antitumor effect; systemic toxicity	[120]
			pH/ROS-responsive drug release			
FDPM	Resistant tumors	Fe_3_O_4_	DHJS inhibits Nrf-2 → overcomes ferroptosis resistance	N/A	Enhanced ferroptosis & antitumor efficacy	[166]
		DHJS (Nrf-2 inhibitor)	Immune modulation in TME			
		Hybrid cell membrane				
rPAE@SPIONs	Solid tumors	SPIONs in pomegranate-like NPs	NIR-triggered DOX release	Mild photothermia	Tumor suppression vs. conventional chemotherapy	[219]
		Reduced poly (β-amino ester)-PEG	Ferroptosis induction			
			M1 macrophage polarization			
PEG-Fe_3_O_**4**_**@C5aRA**	Breast cancer	Hollow Fe_3_O_4_	Blocks C5aR reverses ferroptosis resistance	Metal metabolism regulation	Antitumor efficacy; overcomes ferroptosis resistance	[167]
		C5a receptor antagonist (C5aRA)	M1 macrophage polarization			
AZOSH	Solid tumors	Self-catalytic NO nanocomposite	NO-induced Fe^2+^ overload/GSH depletion ferroptosis	Anti-PD-1	Significant tumor suppression via ICD-induced immunity	[168]
		Arg/H_2_O_2_ reactants	• Facilitates siRNA endosomal escape			
Platelet-engineered “Nanofactory"	Hepatocellular carcinoma (HCC)	Erastin	LOX depletes lactate H_2_O_2_	MRI-guided immunotherapy	Synergistic enhancement of immunotherapy	[169]
		SPIO NPs	Enhanced erastin-induced ferroptosis			
		LOX	Reverses lactate-mediated immunosuppression			

*SAS* sulfasalazine, *GSH* glutathione, *CNPs* chlorin nanoparticles, *DOX* doxorubicin, *NIR* near-infrared, *LOX* lactate oxidase, *ICD* immunogenic cell death, *PDT* photodynamic therapy, *CDT* chemodynamic therapy, *TME* tumor microenvironment

Resistance to ferroptosis represents a significant challenge, which can be addressed through innovative nanoformulations. FDPM nanoparticles counteract Nrf-2-mediated antioxidant defenses by co-delivering Fe₃O₄ and the Nrf-2 inhibitor DHJS. This combination restores ferroptotic susceptibility and enhances immunomodulation, offering a promising solution to ferroptosis resistance [166]. The PEG-Fe₃O₄@C5aRA system blocks C5a/C5aR signaling to reverse ferroptosis resistance while polarizing macrophages toward M1 phenotypes, further boosting antitumor immune responses [167] (Fig. 6C). Advanced platforms integrate ferroptosis with immune stimulation for enhanced therapeutic outcomes. AZOSH, a self-catalytic nitric oxide (NO) nanocomposite, induces Fe²⁺ overload and glutathione depletion to trigger ferroptosis. This system also facilitates siRNA escape, enhancing gene silencing, and, when combined with anti-PD-1 therapy, induces ICD for potent antitumor immunity [168]. Platelet-engineered “nanofactories” that co-load erastin, SPIONs, and lactate oxidase (LOX) deplete immunosuppressive lactate, amplify ferroptosis, and induce ROS-mediated immune activation, with real-time monitoring via MRI [169].

Notably, localized delivery strategies have demonstrated remarkable clinical translation. Intravesical administration of CPBA-modified Fe₃O₄@Chl/Fe CNPs for bladder cancer combines photodynamic therapy (PDT) and CDT to reshape the immunosuppressive TME, resulting in a substantial increase in survival rates from 0–91.7% [170] (Fig. 6D).

Ferroptosis, triggered by IONPs, elevates T-cell infiltration through precise modulation of the TME via the release of immunostimulatory factors and metabolic reprogramming. Upon induction, ferroptotic tumor cells release damage-associated molecular patterns (DAMPs) such as calreticulin (CRT), ATP, and high-mobility group box 1 (HMGB1), which act as “find-me” and “eat-me” signals to recruit dendritic cells (DCs). These DAMPs activate DCs, enabling them to phagocytose tumor antigens released by ferroptotic cells and migrate to lymph nodes for efficient antigen presentation to naive T cells. Concurrently, ferroptosis-driven reactive oxygen species (ROS) accumulation and lipid peroxidation polarize immunosuppressive M2 TAMs toward the pro-inflammatory M1 phenotype, which secrete cytokines like IL-12 and IFN-γ to chemoattract CD8⁺ T cells into the tumor. This cascade—from DAMP-mediated DC activation to cytokine-driven T-cell recruitment—directly enhances T-cell infiltration, as seen in models like GOx@FeNPs, where ferroptosis synergizes with PD-L1 blockade to boost CTL infiltration and achieve > 90% tumor suppression. By bridging ferroptotic cell death with adaptive immune activation, this mechanism strengthens antitumor responses and improves therapeutic outcomes in preclinical settings.

### Reprogramming TAMs via iron oxide nanoplatforms

Building upon initial observations of IONP-mediated macrophage polarization (Sect. "IONPs: intrinsic activators of antitumor immunity"), this section systematizes TAM reprogramming strategies in reversing immunosuppressive microenvironments and activating antitumor immunity (Table 6). One such strategy involves pharmacological inhibition through kinase modulators. For instance, mannose-decorated porous hollow IONPs deliver the PI3Kγ inhibitor 3-MA, which upregulates NF-κB p65 and drives the repolarization of TAMs from the immunosuppressive M2 phenotype to the antitumor M1 phenotype. This approach enhances the secretion of immunostimulatory cytokines, promoting antitumor immunity [171].

**Table 6:** Reprogramming TAMs via iron oxide nanoplatforms

Nanoparticle system	Cancer model	TAM reprogramming Strategy	Key mechanisms	Validation models	Therapeutic outcomes	References
PHNPs@DPA-S-S-BSA-MA@3-MA	Solid tumors	Mannose-targeted delivery of PI3Kγ inhibitor (3-MA)	Upregulates NF-κB p65 pathway	In vitro & in vivo	Immunosuppressive factors; immunostimulatory factors; tumor growth inhibition	[171]
			M2→M1 repolarization			
LPFe₃O₄ NPs	Solid tumors	pH-sensitive PAA coating	Acidic TME-triggered L-Arg release → iNOS/NO production	In vitro & in vivo	Activates T cells; pro-inflammatory cytokines; synergistic tumor elimination	[172]
		L-Arg loading in hollow Fe₃O₄	M2→M1 conversion			
CMPTR/IONPs	B16F10 melanoma	Carboxymethylated β-D-glucan + IONPs	Activates NF-κB/IRF5 pathways	M2-like BMDMs	M1/M2 ratio; CD4^+^/CD8^+^ T-cell infiltration; tumor cell apoptosis	[173]
			M1 polarization	Mouse melanoma model		
Pani/γ-Fe₂O₃ NPs	Breast cancer	Polyaniline-coated γ-Fe₂O₃	CD86^+^ cells	2D IL-10-stimulated macrophages	Successful macrophage uptake; M2→M1 repolarization	[174]
			CD163^+^ cells	3D MCTS		
IOP-enhanced MRI	Osteosarcoma	Clinically translatable IOP nanoparticles	T₂* shortening indicates TAM activation	Rat biodistribution study	Detects CD47 mAb-induced TAM activation	[175]
				Osteosarcoma model		
SPION-CCPMs	ALK + NSCLC	SPION-loaded CCPMs	Reprograms TAMs to pro-inflammatory state	Co-culture with lung cancer cells	Tumor cell proliferation/viability; prevents tumor regrowth after TKI therapy	[27]
				Mouse tumor model		
Ab-decorated IONPs	Subcutaneous/orthotopic tumors	pH-responsive IONP-LSECtin antibody conjugates	Acidic pH-triggered dissociation → IONPs polarize TAMs to M1	Subcutaneous & orthotopic mouse models	CD8^+^ T-cell immunosuppression; significant tumor growth suppression	[176]
			LSECtin blockade on M1 macrophages			
FA-liposomal nanobubbles	NSCLC	Folate-targeted PFH liposomes	LIFU-triggered phase transition Fe₃O₄ release activates IRF5	Not specified in key points	Converts M2→M1 macrophages; T-cell activation/proliferation	[177]
		Fe₃O₄ NPs + STAT3 siRNA	STAT3 siRNA inhibits JAK-STAT3			

PHNPs porous hollow nanoparticles, L-Arg L-arginine, BMDMs bone marrow-derived macrophages, MCTS multicellular tumor spheroids, LIFU low-intensity focused ultrasound, TKI tyrosine kinase inhibitor, PAA polyacrylic acid, iNOS inducible nitric oxide synthase, NO nitric oxide

**Fig. 7:**
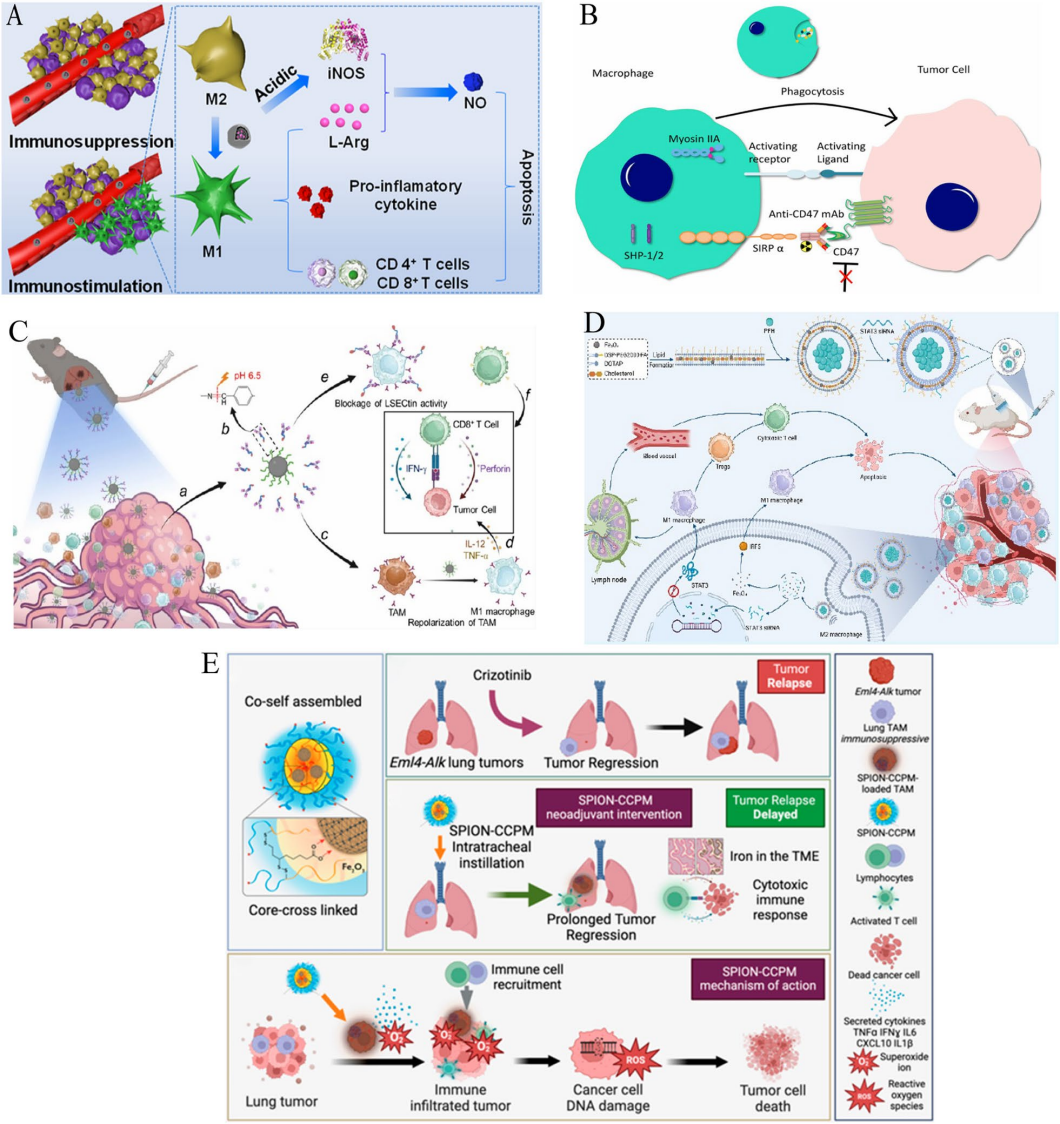
Reprogramming tumor-associated macrophages via iron oxide nanoplatforms. **A** Schematic illustration: Immunomodulation of tumor microenvironment by arginine-loaded iron oxide nanoparticles for gaseous immunotherapy.Reproduced with permission [172].Copyright from American Chemical Society, 2021. **B** Illustrative diagram depicting the mechanism of CD47 monoclonal antibody (mAb)-induced macrophage activation.Reproduced with permission [175].Copyright from Wolters Kluwer Health, Inc. 2024. The interaction between CD47 on osteosarcoma cell surfaces and SIRPα on macrophages inhibits phagocytosis by delivering a strong "don't eat me" signal. The administration of CD47 mAb interrupts these interactions, thereby promoting phagocytosis and facilitating tumor cell clearance. Activation of macrophages subsequently enhances the phagocytic uptake of iron oxide nanoparticles, which can be detected via MRI. **C** Schematic illustration: antibody-decorated nanoplatform to reprogram macrophage and block immune checkpoint lsectin for effective cancer immunotherapy.Reproduced with permission [176].Copyright from American Chemical Society, 2024. **D** Schematic illustration: ultrasound-responsive nanocarriers delivering siRNA and Fe_3_O_4_ nanoparticles reprogram macrophages and inhibit M2 polarization for enhanced nsclc immunotherapy.Reproduced with permission [177].Copyright from American Chemical Society, 2024. E Schematic illustration: SPION-CCPMs reprogram tumor-associated macrophages (TAMs) toward M1 phenotypes and disrupt immunosuppressive niches, preventing ALK-positive NSCLC recurrence after crizotinib cessation.Reproduced with permission [27]. Copyright from American Chemical Society, 2024.

Complementary metabolic strategies exploit the acidic TME to further reprogram TAMs. For example, pH-responsive polyacrylic acid-coated hollow Fe₃O₄ nanoparticles release L-arginine, which is then converted by M1-TAMs into NO, a potent activator of T cells that aids in tumor elimination [172] **(**Fig. 7A**)**. Additionally, carbohydrate-functionalized IONP systems, such as the CMPTR/IONP complexes, activate NF-κB/IRF5 pathways, which enhance the M1/M2 macrophage ratio in melanoma, surpassing the effects of single-agent treatments and boosting CD4⁺ and CD8⁺ T-cell infiltration [173].

In translational models, polyaniline-coated γ-Fe₂O₃ nanoparticles have been shown to increase the proportion of CD86⁺ M1 macrophages in 3D breast cancer spheroids [174]. Moreover, IONPs enhance MRI detection of CD47 monoclonal antibody-induced TAM activation, as evidenced by T₂* shortening in osteosarcoma [175] (Fig. 7B). These findings highlight the potential of IONP-mediated TAM reprogramming for both basic and clinical applications.

Advanced therapeutic platforms integrate dual-pathway targeting for more effective TAM reprogramming. For instance, antibody-conjugated IONPs can release LSECtin-blocking antibodies in the acidic TME, while simultaneously polarizing TAMs to the M1 phenotype, thus alleviating the suppression of CD8⁺ T-cell responses [176] (Fig. 7C). Similarly, folate-targeted lipid nanobubbles co-deliver Fe₃O₄ and STAT3 siRNA. Ultrasound-triggered release of these agents activates IRF5, driving the conversion of M2 to M1 TAMs, while silencing the JAK-STAT3 pathway to block M2 polarization. This dual approach enhances T-cell responses in NSCLC [177] (Fig. 7D).

Additionally, SPION-CCPMs have been demonstrated to reprogram TAMs in ALK-positive lung cancer, delaying tumor growth and preventing recurrence post-therapy [27] (Fig. 7E). These integrated strategies collectively illustrate the potential of IONP-mediated TAM reprogramming as a robust approach to remodel immunosuppressive niches, enhancing the efficacy of conventional immunotherapies.

### Biohybrid nanoplatforms: nature-inspired tumor immunotherapy

Neutrophil-platelet hybrid membranes exemplify this evolution, simultaneously clearing immunosuppressive exosomes and circulating tumor cells [178]. IONP-camouflaged nanovesicles exhibit 3-fold deeper tumor penetration than cell-derived optotheranostics, with sustained tumor retention (168 h vs. 24 h) and synergistic ferroptosis/PDT efficacy—overcoming photobleaching limitations of organic dye-based systems [179]. Innovative biohybrid platforms harness natural biological components to enhance the precision and therapeutic efficacy of IONP-based cancer immunotherapy. One such approach involves engineered platelets that co-deliver anti-PD-L1 antibodies and photothermal IONPs. These platelets adhere specifically to surgical sites, where localized PTT induces necrosis of residual tumor cells. This process not only releases tumor-associated antigens but also blocks immunosuppressive PD-L1 signals, significantly inhibiting postoperative breast tumor recurrence and enhancing CD4⁺/CD8⁺ T-cell infiltration [180].

In a similar strategy, supramolecular conjugates, such as FeAMV, integrate live *Salmonella* VNP20009 bacteria with cancer cell membrane-coated IONPs. This system exploits the bacterial tumor tropism for targeted delivery of ferroptosis inducers, such as Fe₃O₄ and SAS. The resulting ferroptosis (Sect. "Ferroptosis and chemodynamic synergy in iron-enabled cancer immunotherapy") synergizes with bacteria-induced ICD, significantly amplifying antitumor immunity [181].

**Fig. 8:**
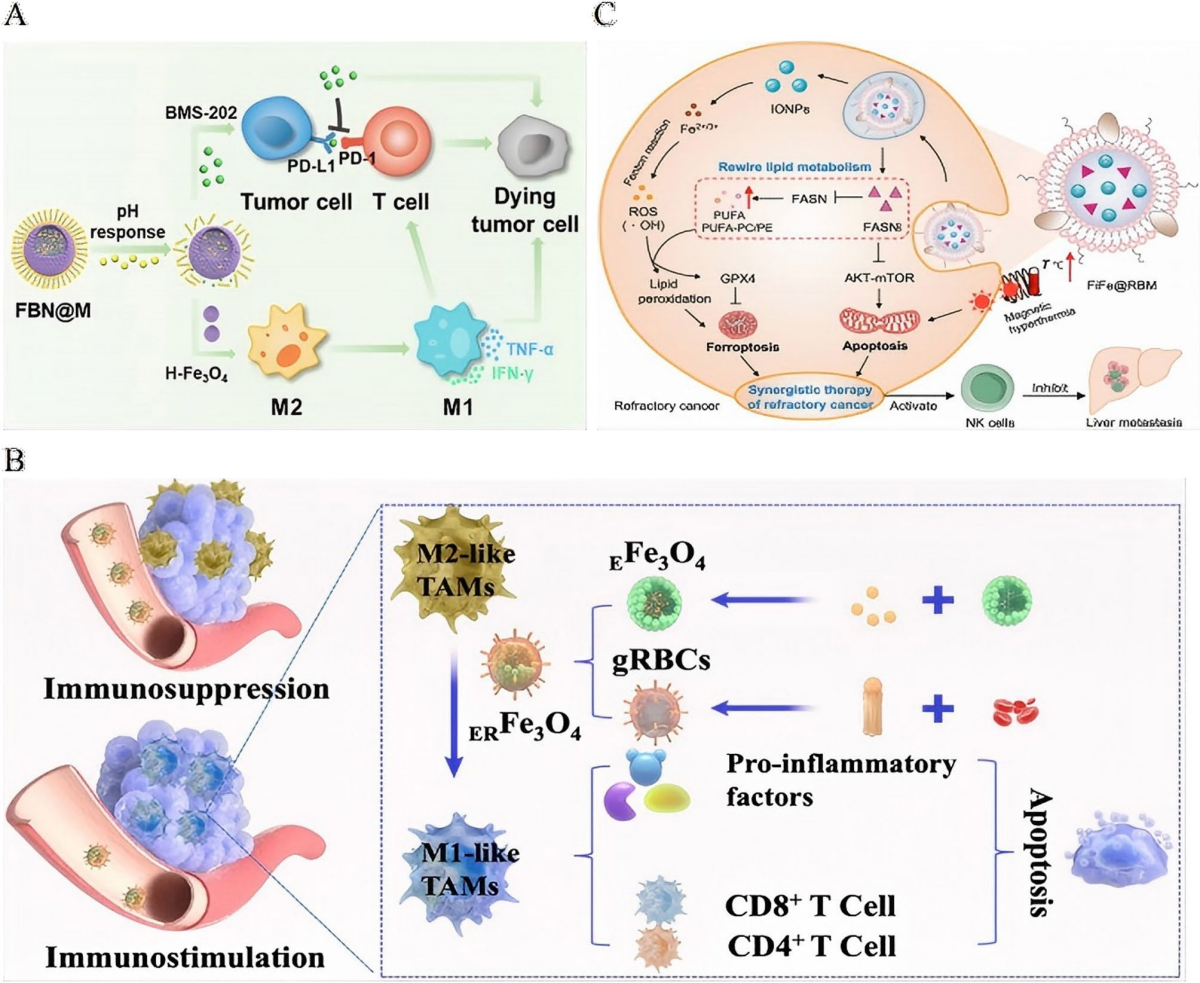
Biohybrid nanoplatforms: nature-inspired tumor immunotherapy. **A** Schematic illustration: construction of hierarchically biomimetic iron oxide nanosystems for macrophage repolarization-promoted immune checkpoint blockade of cancer immunotherapy. Reproduced with permission [182]. Copyright from American Chemical Society, 2024. **B** Schematic illustration: biomimetic iron-based nanoparticles remodel immunosuppressive tumor microenvironment for metabolic immunotherapy.Reproduced with permission [183]. Copyright from Dovepress Taylor & Francis Group, 2024. **C** Schematic illustration: nanovesicles for lipid metabolism reprogram-enhanced ferroptosis and magnetotherapy of refractory tumors and inhibiting metastasis with activated innate immunity. Reproduced with permission [184]. Copyright from American Chemical Society, 2025.

Hierarchical biomimetic designs further optimize therapeutic release through microenvironment-responsive mechanisms. For instance, FBN@M systems encapsulate PD-1/PD-L1 inhibitor BMS-202 and pH-sensitive NaHCO₃ within macrophage membrane-coated hollow IONPs. In response to the acidic TME, CO₂ is generated, which ruptures the membrane and releases BMS-202, while simultaneously reprogramming M2 TAMs into antitumor M1 phenotypes [182] (Fig. 8A).

For metabolic reprogramming, ER Fe₃O₄ nanoparticles employ galactose-modified red blood cell membranes to target M2 TAMs, delivering epiberberine to inhibit lactate production and counteract TME acidosis. Meanwhile, the Fe₃O₄ cores polarize TAMs towards the M1 phenotype, increasing the secretion of pro-inflammatory cytokines (IL-12, IFN-γ, TNF-α), which remodel immunosuppressive niches within the tumor [183] (Fig. 8B).

The precision of these biohybrid and biomimetic systems extends to the suppression of metastasis. FiFe@RBM nanovesicles, which co-encapsulate fatty acid synthase inhibitors and IONPs, selectively accumulate in castration-resistant prostate cancer (CRPC). Here, they induce ROS-mediated mitochondrial dysfunction and inhibit the AKT-mTOR signaling pathway. This dual-action approach promotes both apoptosis and ferroptosis induced by metabolic reprogramming, facilitating MRI-guided magnetothermal eradication of primary tumors. Moreover, the activation of NK cells helps suppress hepatic metastases [184] (Fig. 8C).

### Integrated theranostics and monitoring for precision cancer immunotherapy

IONPs have emerged as key tools in real-time visualization of therapeutic processes and in predicting treatment outcomes, thus bridging diagnostic precision with therapeutic monitoring. One promising approach involves molecular imaging probes such as PDL1-SPIO conjugates, which specifically target PD-L1 on temozolomide-resistant glioblastoma cells. Prussian blue staining, coupled with in vivo T2*-weighted MRI, quantitatively confirms the expression of PD-L1, offering a non-invasive diagnostic method for identifying therapy-resistant gliomas [185]. This capability not only facilitates early diagnosis but also provides valuable insights into the effectiveness of therapeutic interventions.

In addition to diagnostic applications, ultrasmall IONPs have been employed to label adoptively transferred T cells and CAR-T cells, with no observed impairment of their functionality. This approach enables MRI-based spatial mapping of tumor-infiltrating lymphocytes, which serves as a predictive biomarker for therapy response. This technique marks an important step towards personalized immunotherapy, where real-time monitoring of immune cell dynamics is critical for optimizing treatment efficacy [114].

**Fig. 9:**
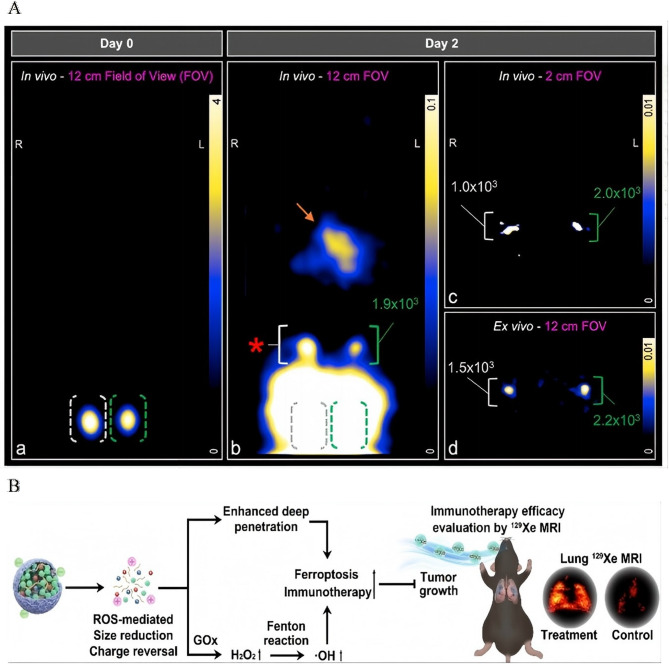
Integrated theranostics and monitoring for precision cancer immunotherapy. **A** Schematic illustration: In vivo tracking of adenoviral-transduced iron oxide-labeled bone marrow-derived dendritic cells using magnetic particle imaging. Reproduced with permission [186]. Copyright from Springer Nature, 2023. **B** Schematic illustration: Monitoring ROS Responsive Fe_3_O_4_-based Nanoparticle Mediated Ferroptosis and Immunotherapy via 129Xe MRI. Reproduced with permission [187]. Copyright from John Wiley and Sons, 2024.

Novel imaging modalities further enhance the capacity for in vivo tracking. MPI addresses the limitations in dynamic range inherent in traditional imaging techniques. By quantifying the migration of SPIO-labeled DCs to lymph nodes in vivo, MPI enables the detection of clinically relevant cell quantities using focused-field scanning. This approach holds significant promise for advancing cellular therapies, providing a more precise and sensitive method for tracking immune cell trafficking during treatment [186] (Fig. 9A).

For therapy-response correlation, ROS-responsive FGTL nanoparticles offer a sophisticated solution for monitoring treatment outcomes. These nanoparticles release GOD and immunostimulatory tuftsin in high-H₂O₂ TMEs, thereby amplifying ferroptosis and promoting the recruitment of effector T cells. This process can be noninvasively monitored using hyperpolarized ¹²⁹Xe MRI, establishing the first radiation-free method for simultaneously inducing ferroptosis and assessing treatment efficacy in metastatic lung cancer. This innovative approach not only enhances therapeutic precision but also provides an essential tool for monitoring dynamic treatment responses [187] (Fig. 9B). Unlike optotheranostic platforms limited by photobleaching and shallow tissue penetration (< 2 cm), IONP-enabled MPI/Xe-MRI overcomes the fundamental trade-off between imaging depth (> 5 cm) and biosafety through radiation-free magnetic detection-a capability unattainable with organic fluorophore-based systems [20].

### IONP-engineered vaccine platforms for amplified antitumor immunity

**Fig. 10:**
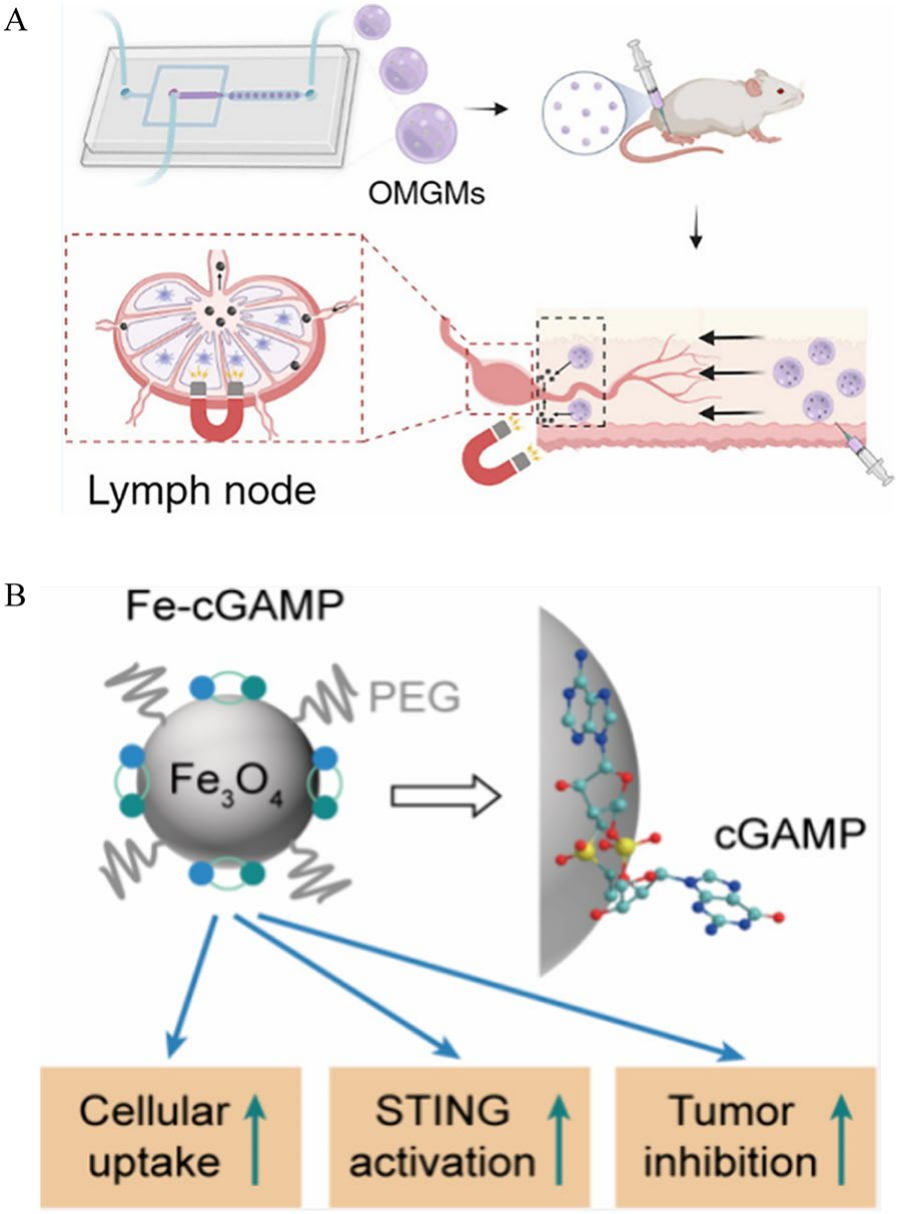
IONP-engineered vaccine platforms for amplified antitumor immunity. **A** Schematic illustration: A Magnetically Driven Biodegradable Microsphere with Mass Production Capability for Subunit Vaccine Delivery and Enhanced Immunotherapy.Reproduced with permission [188]. Copyright from American Chemical Society, 2024. **B** Schematic illustration: Direct cGAMP Delivery via Iron Oxide Nanoparticles for Enhanced STING Activation and Durable Antitumor Immunity. Reproduced with permission [189]. Copyright from American Chemical Society, 2025.

IONPs have emerged as potent platforms for enhancing antigen delivery and immunostimulation in cancer vaccines. One such strategy involves lymph-targeted delivery systems, which leverage magnetic guidance to direct antigen-loaded IONPs to lymph nodes. OVA-loaded IONPs encapsulated in biodegradable gelatin methacrylate microspheres are directed to lymphoid tissues through magnetic navigation. This method enhances both cellular and humoral immunity, resulting in improved antitumor efficacy with a single booster immunization. The sustained release of antigens within the lymph nodes, which are rich in antigen-presenting cells (APCs), promotes a robust immune response [188] (Fig. 10A).

The activation of the STING pathway is another key focus in IONP-based adjuvant design. Acid-ionized iron nanoadjuvants, such as PEIM, co-assemble IONPs with the STING agonist MSA-2. This combination significantly boosts the production of type I interferons (IFN-I) through ROS-dependent NF-κB activation. Furthermore, PEIM enhances lymphatic delivery, promotes cross-presentation by CD169⁺ APCs, and stimulates CD8⁺ T-cell responses. When loaded with autologous tumor membrane antigens, PEIM@Mem vaccines synergize with anti-PD-L1 therapy to effectively prevent postoperative recurrence and metastasis, underscoring their potential in clinical applications [26].

Advancing this approach, Fe-cGAMP conjugates are designed by chemically tethering the STING agonist cyclic GMP-AMP (cGAMP) directly to the surface of IONPs. This configuration not only enhances cellular uptake and STING activation compared to free cGAMP, but also allows IONPs to independently generate ROS and activate Toll-like receptors, resulting in synergistic immune activation. Combining Fe-cGAMP with checkpoint inhibitors has demonstrated remarkable success in inducing complete tumor regression in more than 50% of treated mice, establishing durable antitumor immunity [189] (Fig. 10B).

## Discussion and prospects

IONPs have emerged as versatile and transformative tools in cancer immunotherapy, offering a unique combination of immunomodulation, targeted drug delivery, and theranostic capabilities [133, 190, 191]. Their potential is particularly evident in their ability to reprogram the immunosuppressive TME, a critical challenge in cancer treatment [27, 192, 193]. One of the key features of IONPs is their capacity to influence TAMs, promoting their polarization towards the antitumor M1 phenotype [27, 194, 195]. This shift enhances the immune response against the tumor, providing a promising strategy for overcoming immune evasion by solid tumors. Additionally, IONPs have demonstrated the ability to activate innate immune pathways such as the STING pathway, which plays a crucial role in triggering an effective immune response [26, 189]. These advancements in immune modulation are accompanied by the growing realization of IONPs’ potential to precisely target tumors, improving therapeutic efficacy while reducing off-target effects [94, 95, 196]. Preclinical evidence robustly validates IONPs as transformative immunomodulators. Key successes include: (1) Profound reprogramming of immunosuppressive tumor microenvironments through macrophage polarization and STING pathway activation; (2) Synergistic induction of systemic antitumor immunity when combined with checkpoint inhibitors, achieving complete regression in refractory tumors; (3) Effective metastasis suppression via magnetically targeted immunotherapeutic delivery; and (4) Significant extension of survival across multiple aggressive tumor models through engineered biohybrid platforms. Clinical translation of IONP-based immunotherapies remains predominantly investigational. Current human applications leverage FDA-approved ferumoxytol for diagnostic staging, revealing unique biodistribution patterns in pediatric patients differing from adults: Benign lymph nodes exhibit hypointense hilum and hyperintense parenchyma on T₂-FSE MRI, while malignant nodes show homogeneous hypointensity without discernible hilum. Ferumoxytol accumulation in benign node hila suggests potential as a theranostic bridge for future immune cell priming applications [197].

These preclinical successes are further amplified by the integration of biohybrid designs that leverage cancer cell membranes offer a significant advantage in enhancing the targeting specificity of nanoparticles [198, 199]. By utilizing tumor cell membranes, these biohybrid nanoparticles achieve a level of homologous targeting that allows them to accumulate more effectively in tumor tissues. This approach also significantly extends the systemic circulation time of IONPs, which is crucial for ensuring that the nanoparticles can reach distant tumors before being cleared from the body [80]. Additionally, magnetic navigation has emerged as a powerful tool for enhancing the delivery of therapeutic agents to specific sites, such as lymph nodes, further improving the precision of treatment. The ability of IONPs to combine multiple therapeutic modalities, such as magnetic hyperthermia, PTT, and chemodynamic induction, with immune checkpoint blockade has shown remarkable promise, yielding complete tumor regression in over 50% of preclinical models [104, 133, 152, 189, 200–202]. This convergence of therapeutic strategies exemplifies the potential of IONPs to revolutionize cancer treatment, offering a comprehensive and multifaceted approach to immunotherapy. Unlike metastatic nanovesicles requiring co-encapsulation of 4 + agents, IONP-based theranostics achieve comparable tumor reduction through magnetically amplified Fenton reactions alone, significantly simplifying manufacturing for GMP compliance [179].

A particularly exciting development is the use of engineered immune cells, such as CAR-T and NK cells, labeled with SPIONs [24, 184, 203, 204]. These modified immune cells maintain their effector function while enabling real-time tracking via MRI, which provides valuable insights into their infiltration and activity within the tumor. This ability to monitor immune cell behavior in real time represents a significant step forward in personalized cancer treatment, as it allows clinicians to adjust therapy based on dynamic tumor responses. The combination of MPI and hyperpolarized MRI further enhances the ability to track immune cell recruitment and therapeutic outcomes [205–207]. New correlations have been established between ferroptotic cell death induced by IONPs and the recruitment of immune cells, suggesting that ferroptosis could be leveraged as an immune-stimulating strategy in cancer treatment.

Despite these significant advancements, several challenges remain in translating IONP-based therapies to clinical practice. Tumors often actively develop resistance to IONP-induced immune responses. Golgi apparatus-exosome hybrid nanoparticles represent a pioneering approach to overcome resistance by disrupting PD-L1 trafficking and promoting systemic immunity [208]. For instance, cancer cells can upregulate antioxidant defenses, such as those mediated by the Nrf-2 pathway, to evade ferroptosis and resist the effects of ROS generated by the Fenton reaction [209–212]. Additionally, the accumulation of immunosuppressive metabolites, such as lactate and adenosine, within the TME further impedes the efficacy of immune-based therapies [213–217]. These obstacles highlight the need for more sophisticated approaches to overcome resistance mechanisms and ensure durable therapeutic responses.

Long-term biosafety concerns also persist, particularly regarding the potential for off-target tissue damage caused by ROS generated during IONP-mediated treatments. The risk of accelerated blood clearance upon repeated administration of PEGylated IONPs adds another layer of complexity to their clinical application. Furthermore, the challenges associated with manufacturing these nanoparticles, including batch-to-batch inconsistencies in surface functionalization and difficulties in scaling up biomimetic coating processes, hinder the development of standardized, reproducible products suitable for clinical use. Magnetic targeting efficiency is also a significant limitation, as its effectiveness diminishes in deep-seated or disseminated metastatic tumors. Moreover, the reliance on the EPR effect for passive tumor targeting presents limitations in stroma-rich cancers, such as pancreatic ductal adenocarcinoma, where the tumor vasculature may not exhibit the same degree of permeability. In pediatric cancer patients (*N* = 42), ferumoxytol at 5 mg Fe/kg demonstrated ≤ Grade 2 adverse events in 92% of cases, supporting its acceptable safety profile for diagnostic repurposing [197].

Future research in this field must focus on addressing these challenges and optimizing IONP-based therapies for clinical translation. One promising direction is the development of dynamic, responsive systems that can release therapeutic payloads in response to specific stimuli within the TME, such as proteases or pH and ROS gradients. These advanced systems could allow for more controlled and targeted drug delivery, improving the precision of treatment while minimizing off-target effects. Another exciting avenue is the creation of closed-loop combined imaging/treatment tools that integrate real-time immune monitoring with feedback-controlled therapy. By combining MPI or MRI with AI-guided therapy adjustments, such systems could enable personalized, adaptive treatment regimens based on tumor progression and immune response.

Additionally, the application of synthetic biology offers the potential for even more innovative approaches, such as the development of tumor-colonizing IONP-bacteria hybrids. These bacteria could serve as carriers for IONPs, delivering them specifically to tumor sites, while genetically encoded nanobodies displayed on the surfaces of IONPs could enhance tumor targeting and immune modulation. To facilitate the clinical translation of these technologies, it is crucial to establish good manufacturing practice (GMP)-compliant production methods for IONP-immune cell conjugates. Optimizing synergy between ferroptosis inducers (Sect. "Ferroptosis and chemodynamic synergy in iron-enabled cancer immunotherapy'') and immune checkpoint inhibitors, and validating the efficacy of these therapies in patient-derived organoids, will also be key steps in moving towards clinical implementation.

## Conclusion

Iron oxide nanoparticles (IONPs) have emerged as transformative theranostic tools in preclinical cancer immunotherapy, driving breakthroughs across immune modulation, targeted delivery, and multimodal therapeutic synergy. Their core strength lies in reprogramming the immunosuppressive tumor microenvironment (TME): IONPs polarize M2-polarized tumor-associated macrophages (TAMs) to antitumor M1 phenotypes, activate innate immune pathways like STING, and induce immunogenic ferroptosis, converting “immune-cold” tumors into inflamed, treatment-responsive states.

Complemented by precision delivery strategies—including utilization of the enhanced permeability and retention (EPR) effect, magnetic navigation, and cancer cell membrane-mediated homologous targeting—IONPs achieve selective tumor accumulation while minimizing off-target toxicity. For instance, biohybrid systems like SPIO NP@M-P extend the half-life of immunomodulatory peptides by 60-fold, amplifying T-cell reactivation. Preclinical models validate striking outcomes: combinatorial IONP-based regimens integrating immune checkpoint blockade, photothermal/magnetic hyperthermia, or chemodynamic therapy have achieved over 50% complete tumor regression in aggressive models, with > 90% suppression in colorectal cancer via ferroptosis-immunotherapy synergy. Engineered immune cells, such as magnetically guided NK cells and IONP-labeled CAR-T cells, retain effector function while enabling real-time MRI monitoring of tumor infiltration, bridging efficacy with theranostic precision.

Beyond direct tumor killing, IONPs enhance systemic immunity by promoting dendritic cell maturation, CD8⁺T-cell infiltration, and durable antitumor memory, addressing limitations like checkpoint inhibitor resistance. Their versatility is further amplified by integrated diagnostics, including MRI/magnetic particle imaging (MPI)-guided monitoring of immune cell trafficking and treatment responses, enabling personalized adjustments. While challenges such as adaptive resistance and scalable manufacturing persist, these preclinical breakthroughs position IONPs as cornerstones of next-generation precision oncology. Realizing their full potential will require advancing nanoparticle design, manufacturing, and clinical testing—efforts that can pave the way for more effective, personalized, and safe cancer treatments by deepening understanding of TME, immune modulation, and targeted delivery.

## Data Availability

No datasets were generated or analysed during the current study.

## Abbreviations

IONPs: Iron oxide nanoparticles
MRI: Magnetic resonance imaging
EPR: Enhanced permeability and retention
TME: Tumor microenvironment
MPI: Magnetic particle imaging
TAMs: Tumor-associated macrophages
MDSCs: Myeloid-derived suppressor cells regulatory T cells
Tregs: Transforming growth factor-β
NK: Natural killer
EGFR: Epidermal growth factor receptors
CAR: Chimeric antigen receptor
gRNA.: Guide RNA.
RNP: Ribonucleoprotein
ROS: Reactive oxygen species
OVA: Ovalbumin
LPS: Lipopolysaccharide
IONP-COOH: Carboxymethyl dextran-coated IONPs
AML: Acute myeloid leukemia
FH-MPLA: Ferumoxytol-loaded monophosphoryl lipid A
ICG: Co-loaded indocyanine green
NIR: Near-infrared
ICD: Immunogenic cell death
NSCLC: Non-small cell lung cancer
CMNPs: Nanoparticles with cancer cell membranes
FVIOs: Ferromagnetic vortex-domain iron oxide nanorings
IONAs: Iron oxide nanoparticle assemblies
PTT: Photothermal therapy
HCSVs: Hybrid vesicles
CHINPs: Cancer cell membrane-camouflaged nanoplatforms
SPIO: Superparamagnetic iron oxide
SDT: Sonodynamic therapy
PpIX: Protoporphyrin IX
DCs: Dendritic cells
SPIONs: Superparamagnetic iron oxide nanoparticles
MINPs: Magnetically responsive immunostimulatory nanoreagents
ODNs: Oligodeoxynucleotides
SAS: Sulfasalazine
IONVs: Iron oxide nanovaccines
GOD: Glucose oxidase
CDT: Chemodynamic therapy
ART: Artemisinin
NO: Nitric oxide
LOX: Lactate oxidase
PDT: Photodynamic therapy
CRP: Castration-resistant prostate cancer, C
IFN-I: Type I interferons
APCs: Antigen-presenting cells
cGAMP: Cyclic GMP-AMP
